# Molecular Mechanism of *ZjWRKY40‐zju‐miR157* Module Regulating Phytoplasma Tolerance in Jujube

**DOI:** 10.1111/mpp.70219

**Published:** 2026-02-13

**Authors:** Changfeng Ai, Lixin Wang, Zhi Luo, Yunjie Wang, Xuan Zhao, Lili Wang, Jiaqi Sun, Kunyi Lv, Xueqing Yan, Haonan Cao, Noor Muhammad, Qiong Zhang, Mengjun Liu, Zhiguo Liu

**Affiliations:** ^1^ College of Horticulture Hebei Agricultural University Baoding Hebei China; ^2^ College of Horticulture and Forestry Tarim University Alar Xinjiang China; ^3^ College of Forestry Hebei Agricultural University Baoding Hebei China; ^4^ Shandong Institute of Pomology Tai'an Shandong China; ^5^ Research Center of Chinese Jujube Hebei Agricultural University Baoding Hebei China

**Keywords:** phytoplasma, SJP4_JWB_, *ZjSPL3*, *zju‐miR157*, *ZjWRKY40*

## Abstract

Phytoplasma (‘*Candidatus* Phytoplasma’), a bacterial pathogen, is a significant plant health concern worldwide, resulting in substantial economic losses. In jujube ( *Ziziphus jujuba* ), it is often referred to as a cancer‐like disease that can destroy the whole plant and cause great economic loss, posing serious challenges to jujube's sustainable development. Here, the phytoplasma tolerance mechanism in jujube was revealed by the identification of a highly phytoplasma‐tolerant jujube genotype. Integrated transcriptomic and metabolomic analyses were conducted to compare a phytoplasma‐susceptible genotype Fuxiang with a tolerant genotype T13. The results revealed that the *ZjWRKY40* gene was significantly upregulated in T13 about 3‐fold at the third development stage, suggesting its key role in mediating phytoplasma tolerance. A phytoplasma effector SJP4_JWB_ was identified and shown to interact with ZjWRKY40 by yeast two‐hybrid and co‐immunoprecipitation methods. In addition, ZjWRKY40 was found to bind to the promoter of *zju‐miR157*, thereby regulating its expression. Moreover, *zju‐miR157* targeted *ZjSPL3* and negatively affected the phytoplasma tolerance by downregulating *ZjSPL3* expression. Together, these findings outline a regulatory network involving SJP4_JWB_ *–ZjWRKY40–zju‐miR157*, which provides important insights into the molecular mechanism of the phytoplasma tolerance in jujube and lays a foundation for developing tolerant genotypes through molecular breeding.

## Graphical abstract

SJP4_JWB_ regulated the ZjWRKY40‐zju‐miR157 module to modulate the differential phytoplasma tolerance between phytoplasma‐susceptible genotype Fu and phytoplasma‐tolerant genotype T13.

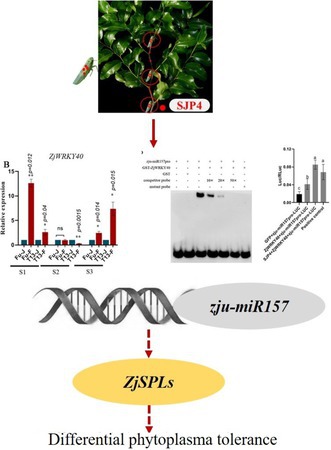

## 1 Introduction

Phytoplasma diseases are a pervasive threat to global agriculture, causing widespread destruction across a broad spectrum of economically vital crops and inflicting substantial yield and economic losses (Bertaccini et al. 2014; Wang, Bai, et al. 2024). For example, an outbreak of phytoplasma disease named Côte d'Ivoire Lethal Yellowing (CILY) has destroyed over 350 ha of coconut plantations and poses an ongoing threat to a further 7000 ha (Gurr et al. 2016). In jujube (*Ziziphus jujuba*), phytoplasma disease kills at least 3%–5% of all the jujube trees each year in many orchards (Zhao et al. 2019). Jujube witches' broom (JWB), associated with the presence of phytoplasmas, poses a major barrier to the sustainable cultivation of jujube, not only in China (Tian et al. 2002) but also in other countries like Japan (Ohashi 1996) and Korea (Jung et al. 2012). JWB leads to excessive shoot proliferation and malformation of flowers (Tian 1998), diminishes fruit quality (Tian 1998), and can cause yield reductions between 30% and 80% (Wang et al. 2015). In severe cases, infected trees may die within 3–5 years of initial infection (Tian et al. 2002; Liu and Zhao 2008). A survey conducted in various jujube‐producing regions of China revealed that the incidence of JWB ranged from 18% to 84%, the rate depending on the planting region and cultivar (Tian et al. 2002). JWB is considered as the cancer of jujube, posing a severe threat to its sustainable production (Wang et al. 2007; Ma et al. 2023; Zhou et al. 2021; Wang, Liu, et al. 2022).

Phytoplasmas (‘*Candidatus* Phytoplasma’) are a unique group of cell‐wall‐less, phloem‐restricted, bacteria within the class *Mollicutes*. They are transmitted by insect vectors and associated with symptoms such as witches' broom, phyllody, yellowing, leaf shrinkage and stunted growth (Bertaccini et al. 2014; Zhao et al. 2019; Oshima et al. 2023). The phytoplasmas are mainly transmitted by phloem feeders, especially leafhoppers, which spread them by piercing‐sucking mouthparts (Suzuki et al. 2024). During infection, phytoplasma‐secreted effectors manipulate host cellular functions (Oshima et al. 2023; Ma, Huang, et al. 2024). Among these, effector proteins such as SAP05, SAP11, SAP54 and TENGU have been identified, and their roles in inducing morphological changes have been characterised (Oshima et al. 2023). SAP05 induces more extensive and dramatic symptoms than SAP11 and SAP54 in transgenic *Arabidopsis thaliana* . Mutation of the plant RPN10 amino acid sequence confers tolerance to phytoplasma pathogens, specifically those secreting SAP05 (Huang et al. 2021; Furch et al. 2021). In *Paulownia*, the effectors Pawb 3/9/16/37/51 target *PfSPL1*, whose stability is regulated by PfmiR156; this leads to the degradation or modulation of PfSPL1 contributing to the witches' broom symptoms (Yang, Wang, et al. 2023). In wheat, the effector SWP12 targetes and destabilises the transcription factor TaWRKY74, reducing the expression of defence‐related genes and increasing susceptibility to ‘*Ca*. Phytoplasma tritici’ infection (Bai et al. 2022). In jujube, the phytoplasma effector SJP1/2 alters the *ZjBRC1*‐mediated abscisic acid pathway to induce lateral bud development (Ma et al. 2023). In addition, SJP1/2 also interact with ZjTCP2, which is regulated by *miRNA319f‐1*, leading to leaf shrinkage. Moreover, SJP1/2 destabilise *ZjTCP7‐ZjFD* modules, downregulating *ZjYUCCA2* expression, thus decreasing the auxin levels and promoting shoot branching (Ma, Huang, et al. 2024).

Regarding phyllody formation, effector SJP3 interacts with and degrades MADS‐box protein SHORT VEGETATIVE PHASE 3 (ZjSVP3), which regulates the expression of *ZjZFP8* and *ZjSHP1*, ultimately inducing pistil reversion in jujube (Deng et al. 2024). PHYL1_JWB_ facilitates the proteasome‐mediated degradation of essential flower morphogenetic regulators to induce aberrant floral development under phytoplasma infection (Xue et al. 2024). The *ZjERF18–zju‐miR156c–ZjSPL3* module mediates cytokinin accumulation and plays a critical role in JWB symptom development (Wang, Luo, et al. 2024). The effector Zaofeng6 interacts with ZjTCP7 and downregulates its expression to induce JWB symptoms (Chen et al. 2022), while *ZjTCP16* expression is induced by phytoplasma infection and its overexpression in *A. thaliana* and jujube leads to dwarfism and small leaves (Yang et al. 2022). Multiple effectors have been shown to specifically target TCPs, and suppression of TCP activities represents a novel strategy for phytoplasma prevention (Correa Marrero et al. 2024). Phyllogens, another class of phytoplasma effectors, interact with MADS‐box transcription factors (MTFs) and the RAD23 complex to mediate their degradation via the host proteasome, resulting in phyllody (Suzuki et al. 2024; Kitazawa et al. 2022). Despite significant advances in understanding how phytoplasmas manipulate host growth and development, relatively few studies have explored the molecular mechanisms leading to phytoplasma tolerance in plants.

Jujube is the most widely cultivated fruit tree in China and has also been introduced to over 40 countries. Over 7000 years of natural and human‐driven domestication have endowed jujube with unique traits (Liu et al. 2020; Wang et al. 2025). It is highly nutritious, especially rich in cyclic AMP (cAMP), which is beneficial to human health (Wang et al. 2023; Muhammad et al. 2024), and is tolerant to drought and salinity stresses (Ma et al. 2022). Unlike in other species, phytoplasma‐tolerant genotypes within the *Ziziphus* genus have been identified using methods such as grafting or phytoplasma‐infected branch bark inoculation (Wang, Liu, et al. 2022; Xu et al. 2023), and their tolerance mechanisms have been partially reported. For example, *Ziziphus mauritiana* exhibits tolerance to phytoplasma, which is linked to cinnamoyl‐CoA reductase‐like *SNL6*. The *ZjMYB44‐ZjPOD51* module could improve the tolerance to phytoplasma in jujube (Xu et al. 2023; Zhang et al. 2025). Chemical control methods—including insecticides and antibiotics—are currently widely employed but negatively affect plant health and the environment. The scarcity of naturally tolerant germplasm hinders the development of sustainable, long‐term phytoplasma management strategies. However, encouragingly, recent studies have identified highly phytoplasma‐tolerant genotypes (Wang, Liu, et al. 2022), underscoring the importance of unravelling the molecular basis of this trait. Existing research has largely relied on homology‐based gene cloning, identification or expression profiling, with limited functional validation (Wang, Liu, et al. 2022). To address this gap, the current study conducted integrated transcriptomic, metabolomic and gene functional verification analyses comparing a phytoplasma‐susceptible genotype, Fuxiang (Fu), with a tolerant genotype, T13. Our study aimed to elucidate the molecular mechanisms underlying phytoplasma tolerance in jujube. The findings not only advance a comprehensive understanding of tolerance pathways but also provide key insights for developing phytoplasma‐resistant genotypes capable of suppressing pathogen load and disrupting the transmission cycle—vital components of sustainable, preventive disease management strategies.

## 2 Results

### 2.1 Phenotypes of Susceptible and Tolerant Jujube Genotypes Under Phytoplasma Infection

**FIGURE 1:**
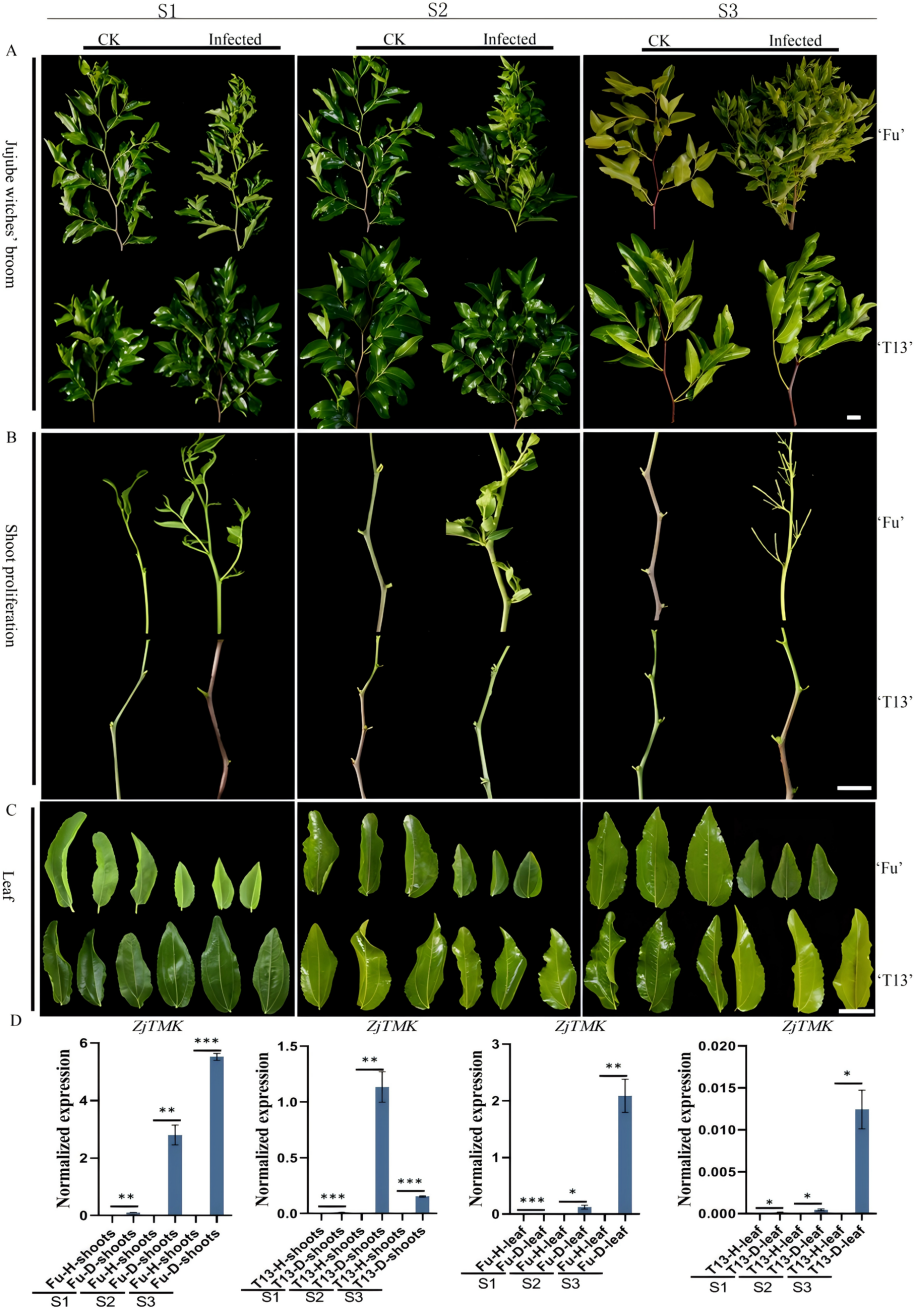
The whole phenotype observation (A), shoot proliferation (B) and leaf (C) changes in susceptible genotype Fuxiang (Fu) and tolerant one T13 under phytoplasma infection at three growth stages (S1–S3), while both uninfected genotypes were taken as control (CK). S1–S3 represented three growth stages. (D) The phytoplasma detection by the expression of *ZjTMK* analysis in Fu and T13 genotypes under infected and healthy plants. H stands for healthy, and D stands for diseased. *, ** and *** indicated the significant difference at *p* < 0.05, *p* < 0.01 and *p* < 0.001 level, respectively.

To investigate the effects of phytoplasma infection on the susceptible genotype Fu and the tolerant genotype T13, scions of each were grafted onto healthy and diseased Fu rootstocks. Their phenotypes were then observed across three growth stages over one year (S1–S3). As shown in Figure 1, at the S1 stage, both genotypes showed jujube witches' broom symptoms compared to healthy plants, but the phenotype of T13 was not as severe as that of Fu. Specifically, axillary bud germination, shoot proliferation and smaller leaves were observed in Fu plants compared to the healthy control, while only axillary bud germination was observed in T13 plants (Figure 1B,C).

At the S2 stage, more severe jujube witches' broom symptom was observed in Fu plants with typical phyllody, witches' broom, and smaller leaves (Figure 1A–C). However, the jujube witches' broom symptom was rescued in T13 plants, which showed normal shoots and leaves. By the S3 stage, the jujube witches' broom symptom became more severe in Fu plants, whereas the infected T13 plant grew asymptomatic (Figure 1A–C).

To verify the content of phytoplasma in both genotypes, the expression level of *ZjTMK* was tested in shoots and leaves. As shown in Figure 1D, in both shoots and leaves of infected Fu, the expression level of *ZjTMK* significantly increased from the S1 to S3 stage; its expression level was significantly higher in shoots than in leaves. In T13, the expression level of *ZjTMK* increased in shoots at the S2 stage and in leaves at the S3 stage. However, compared to Fu shoots and leaves at three growth stages, the expression level in T13 plants remained at a lower level from stage S2 to stage S3. These results collectively demonstrate that Fu is a phytoplasma‐susceptible genotype, while T13 is a phytoplasma‐tolerant genotype.

### 2.2 Transcriptome Profiles in Susceptible and Tolerant Jujube Genotypes Under Phytoplasma Infection

Transcriptome sequencing was performed to elucidate the molecular mechanism behind the difference between Fu and T13 under phytoplasma infection at three growth stages. A total of 263.57 Gb of high‐quality reads was obtained, with a Q30 base percentage exceeding 92.87%, indicating strong sequencing accuracy. The clean reads were aligned with the jujube reference genome, with alignment rates ranging from 88.03% to 90.24% (Table S2). In addition, principal component analysis (PCA) showed that the samples clustered into distinct regions along PC1 and PC2. Pearson correlation analysis also revealed high similarity among replicates (Figure S1). These results indicate the reliability and reproducibility of the RNA‐seq data. Differentially expressed genes (DEGs) were identified in different comparable groups in both genotypes to elucidate the transcriptional changes under phytoplasma infection. We identified 2287, 1971 and 7387 DEGs in Fu at the three growth stages: 1164 upregulated and 1123 downregulated at the S1 stage, 859 upregulated and 1112 downregulated at the S2 stage, and 6007 upregulated and 1380 downregulated at the S3 stage. W 2771, 2387 and 1798 DEGs were identified in T13 at the three growth stages, respectively. At the S1 stage, 1043 genes were upregulated and 1728 were downregulated. At the S2 stage, 978 were upregulated and 1409 were downregulated. At the S3 stage, 1450 genes were upregulated, while 348 were downregulated (Figure S1 and Table S3). In addition, hierarchical clustering heatmap analysis revealed more distinct transcriptomic changes in T13 during the early stages (S1 and S2) compared to Fu (Figure S1; Tables S4 and S5). Conversely, Fu exhibited a larger number of DEGs (4972) at the S3 stage compared to T13 (974 DEGs) (Figure S2). Moreover, Gene Ontology (GO) enrichment analysis was performed on stage‐specific DEGs in both genotypes. At the S1 stage, DEGs in Fu were primarily enriched in processes such as regulation of DNA methylation and D‐gluconate catabolic process. In contrast, DEGs in T13 were mainly associated with the abscisic acid‐activated signalling pathway and response to oxygen‐containing compounds. At the S2 stage, DEGs in Fu and T13 were mainly enriched in microtubule‐based movement, polysaccharide biosynthetic process, sucrose biosynthetic process and terpenoid biosynthetic process, respectively. At the S3 stage, DEGs in Fu and T13 were mainly enriched in carbohydrate derivative catabolic process, aminoglycan metabolic process and response to stimulus, protein phosphorylation, respectively (Figure S1).

**FIGURE 2:**
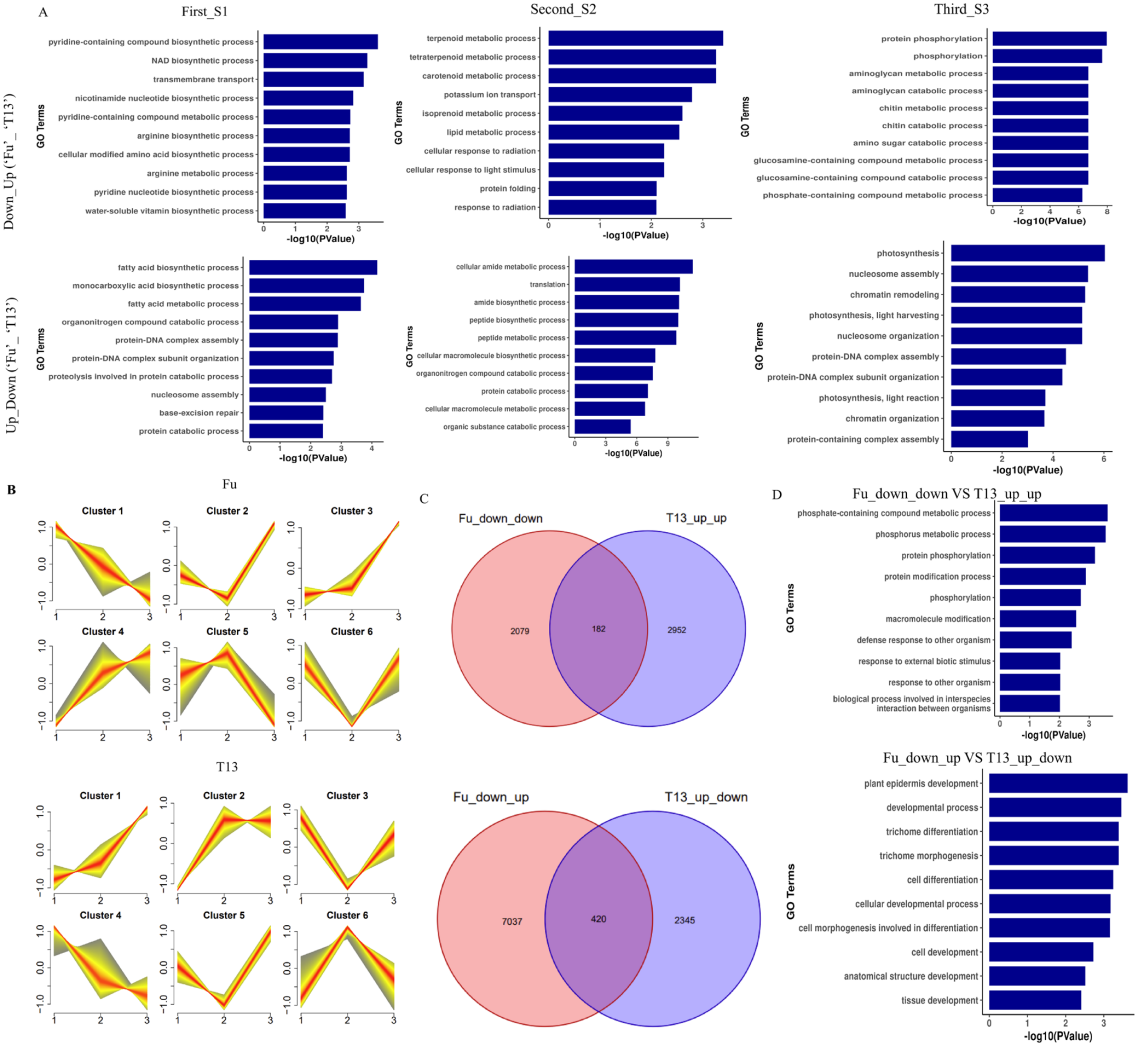
The expression patterns of differentially expressed genes (DEGs), Venn diagrams and GO enrichment analysis. (A) GO enrichment of biological processes in Down_Up and Up_Down (Fu_ T13) groups at three growth stages, S1, S2 and S3. (B) Six clusters were generated according to the expression patterns of DEGs in Fu and T13 genotypes. The figures 1 to 3 on the *x*‐axis represent the three growth stages, S1, S2 and S3. (C) Venn diagrams of two comparison groups: Fu_down_down versus T13_up_up and Fu_down_up versus T13_up_down. Among them, Fu_down_down versus T13_up_up represented Fu (Cluster1) and T13 (Cluster1); Fu_down_up versus T13_up_down represented Fu (Cluster2+Cluster6) and T13 (Cluster6). (D) GO enrichment analysis of the common DEGs from Fu_down_down versus T13_up_up and Fu_down_up versus T13_up_down groups.

To further characterise genotype‐specific responses, scatterplots of DEGs were generated to compare Fu and T13 at each stage (Figure S3). DEGs that were downregulated in Fu but upregulated in T13 (or vice versa) were identified and subjected to GO enrichment analysis. Pyridine‐containing compound biosynthetic process, fatty acid biosynthetic process, terpenoid metabolic process, cellular amide metabolic process, protein phosphorylation and photosynthesis were enriched in Down_up and Up_down (Fu_ T13) groups at the three growth stages (Figure 2A and Table S6). Next, the cluster analysis of DEGs with the k‐means clustering algorithm was performed. As shown in Figure 2B, six clusters were categorised in both genotypes with different expression patterns at three growth stages in Fu and T13 (Table S7 and Figure S4). We mainly focused on the common DEGs between the up_up changing pattern in T13, the down_down changing pattern in Fu and the up_down changing pattern in T13, the down_up changing pattern in T13. A total of 182 and 420 common DEGs were identified in the above comparisons, respectively (Figure 2C). Gene Ontology (GO) enrichment analysis of the Fu_down_down versus T13_up_up group revealed significant enrichment in phosphate‐containing compound metabolism, protein phosphorylation, defence responses to organisms and interspecies interactions. In contrast, the Fu_down_up versus T13_up_down group was enriched in processes related to epidermis development, trichome differentiation, cell morphogenesis and cellular development (Figure 2D; Table S8). These findings suggest that the transcriptional regulation of key biological processes, including phosphorylation, metabolite biosynthesis and cell differentiation, may contribute to the differential phytoplasma tolerance observed between the Fu and T13 genotypes.

### 2.3 Metabolomic Alterations in Susceptible and Tolerant Jujube Genotypes Under Phytoplasma Infection

To investigate the metabolite‐level responses associated with the phytoplasma tolerance, the susceptible genotype Fu and tolerant genotype T13 were subjected to widely targeted metabolome analysis using a UPLC‐ESI‐MS/MS system. PCA and Pearson correlation analysis demonstrated that the samples in each group clustered together and exhibited high correlation, confirming the reliability of the data (Figure S5). In addition, the samples of healthy (J) and phytoplasma‐infected (F) samples in Fu were separated into two groups, whereas this separation was less pronounced in T13, especially for the third growth stage (S3). This suggests a greater degree of differentially accumulated metabolites (DAMs) between healthy and infected tissues in Fu than in T13.

A total of 232 metabolites were identified through KEGG and HMDB‐based annotation, including 64 carboxylic acids and derivatives, 35 fatty acyls, 22 organooxygen compounds, 11 prenol lipids and 10 benzene and substituted derivatives (Figure S6). Using the thresholds of VIP ≥ 1 and log_2_FC ≥ |1|, different numbers of DAMs were identified in the phytoplasma‐infected plants compared to their healthy control. In Fu, a total of 268, 337 and 394 DAMs were identified at the S1, S2 and S3 growth stages, respectively. At the S1 stage, 165 DAMs were upregulated and 103 were downregulated. At the S2 stage, 205 were upregulated and 132 downregulated, while at the S3 stage, 184 DAMs were upregulated and 210 were downregulated. Similarly, 392, 366 and 365 DAMs were identified in T13 at the three growth stages, respectively, including 198 upregulated and 194 downregulated at the S1 stage, 194 upregulated and 172 downregulated at the S2 stage, 294 upregulated and 171 downregulated at the S3 stage (Figure S5 and Tables S9 and S10). Hierarchical clustering heatmap analysis showed the trend of the metabolites in the two genotypes at the three growth stages (Figures S5 and S7). Among them, more drastic changes were detected in T13 at the S3 growth stage compared to those in Fu (Tables S11 and S12). All these results demonstrated that distinct metabolite accumulating patterns exist between Fu and T13 genotypes under phytoplasma infection.

**FIGURE 3:**
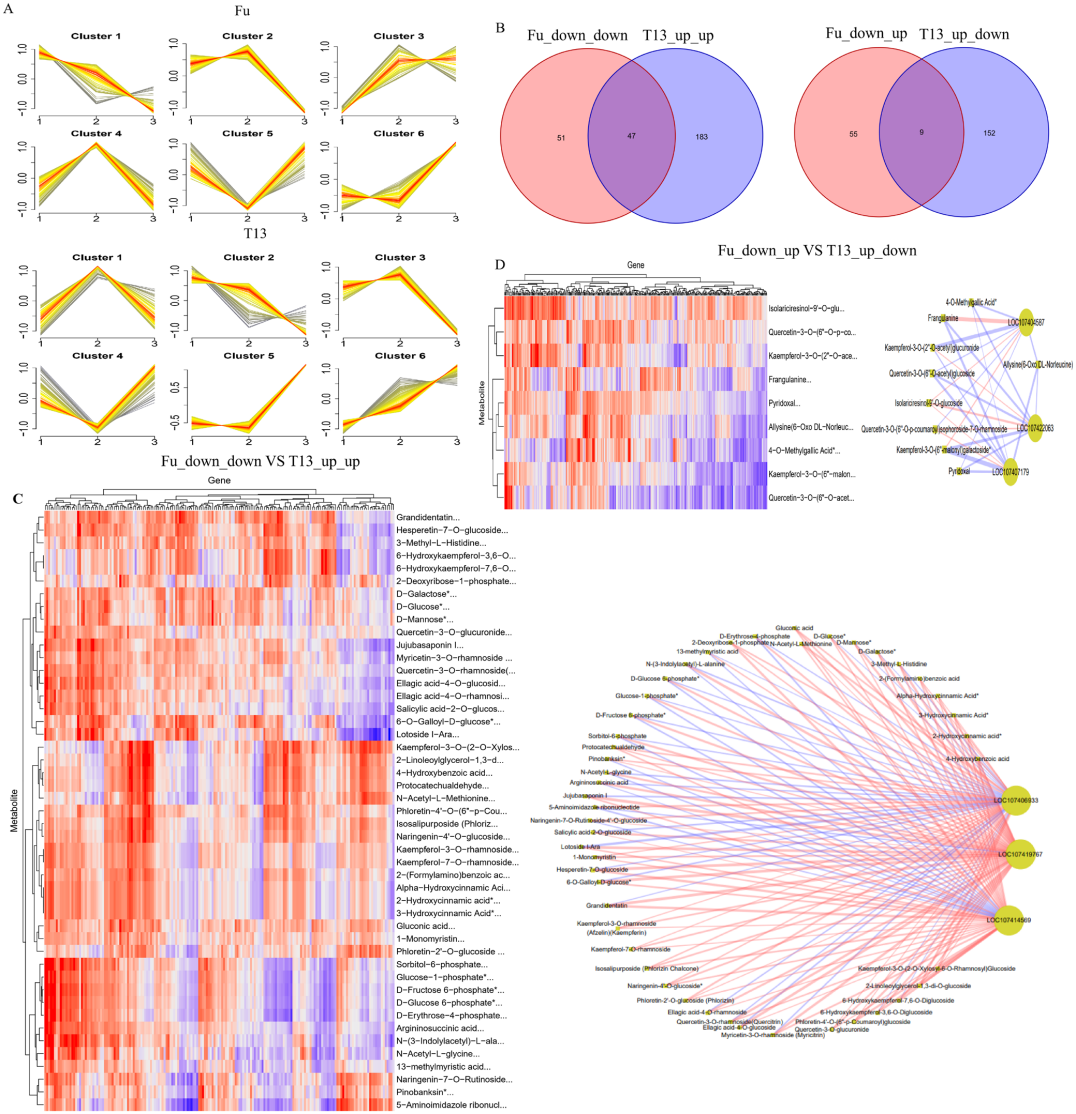
Metabolic alterations and correlation analysis between transcriptome and metabolome in Fu_down_down versus T13_up_up and Fu_down_up versus T13_up_down groups, respectively. (A) Six clusters were generated according to the changing patterns of differentially accummulated metabolites (DAMs) in Fu and T13 genotypes. The figures 1 to 3 on the *x*‐axis represent the three growth stages, S1, S2 and S3. (B) Venn diagram analysis of two comparison groups: Fu_down_down versus T13_up_up and Fu_down_up versus T13_up_down. Among them, Fu_down_down versus T13_up_up represented Fu (Cluster1) and T13 (Cluster5 + Cluster6), Fu_down_up versus T13_up_down represented Fu (Cluster5) and T13 (Cluster1 + Cluster3). (C) Pearson correlation heatmap and network analysis between related differentially expressed genes (DEGs) and DAMs in Fu_down_down versus T13_up_up group. (D) Pearson correlation heatmap and network diagram between related DEGs and DAMs in Fu_down_up versus T13_up_down group. In the network diagram, ellipses represent genes and rectangles represent metabolites. The red nodes represent genes or metabolites with an upward trend, while the blue nodes represent genes or metabolites with a downward trend. The connections between nodes represent correlation, and the thickness represents the degree of the connections. The red connections represent a positive correlation, and the blue connections represent a negative correlation.

To identify key metabolites involved in differential phytoplasma tolerance, DAMs were categorised into six clusters in each genotype (Figure 3A). Each cluster displayed unique temporal trends. For instance, Cluster 1 metabolites in Fu increased steadily from S1 to S3 stages, while the same cluster in T13 showed an increase followed by a decrease. DAMs associated with each cluster are listed in Table S13. However, among the clusters, it was primarily focused on the common DAMs between the up_up change pattern in T13, such as Cluster 5 and Cluster 6, and the down_down change pattern in Fu, such as Cluster 1. In addition, the up_down change pattern (Cluster 1+Cluster 3) in T13 and the down_up change pattern (Cluster 5) in T13 were compared. Venn diagram analysis identified 47 DAMs shared in the Fu_down_down versus T13_up_up group, and 9 DAMs in the Fu_down_up versus T13_up_down group (Figure 3B). Among the 47 shared DAMs, several key metabolite classes were identified. These included flavonoids and their glycosides, such as isosalipurposide, phlorizin chalcone and naringenin‐7‐*O*‐rutinoside‐4′‐*O*‐glucoside. Phenolic acids and their derivatives were also present, including 3‐hydroxycinnamic acid and salicylic acid‐2‐*O*‐glucoside. Additionally, various sugars and their derivatives were found, such as d‐fructose 6‐phosphate, sorbitol‐6‐phosphate, d‐glucose 6‐phosphate, d‐glucose, d‐mannose and d‐galactose. Other organic compounds included gluconic acid, argininosuccinic acid and 13‐methylmyristic acid. Furthermore, nine DAMs were specifically detected in the Fu_down_up versus T13_up_down group. These included kaempferol‐3‐*O*‐(2″‐*O*‐acetyl) glucuronide, frangulanine, kaempferol‐3‐*O*‐(6″‐malonyl) galactoside, pyridoxal, quercetin‐3‐*O*‐(6″‐*O*‐acetyl) glucoside, isolariciresinol‐9′‐*O*‐glucoside, allysine (6‐oxo‐dl‐norleucine), 4‐*O*‐methylgallic acid and quercetin‐3‐*O*‐(6″‐*O*‐*p*‐coumaroyl) sophoroside‐7‐*O*‐rhamnoside (Table S14). In addition to these, several important DAMs were found in other comparison groups between Fu and T13. These included ferulic acid, γ‐aminobutyric acid (GABA), indole‐3‐acrylic acid, l‐asparagine and *N*,*N*‐dimethylglycine (vitamin B16). The differential biosynthesis of these metabolites may contribute to the phytoplasma tolerance observed in T13 compared to Fu.

### 2.4 Correlation Analysis Between Transcriptome and Metabolome Alterations Under Phytoplasma Infection

To explore the relationship between DEGs and DAMs, we performed a correlation analysis integrating transcriptomic and metabolomic data. This analysis focused on two comparison groups: Fu_down_down versus T13_up_up and Fu_down_up versus T13_up_down. In the Fu_down_down versus T13_up_up group, a total of 47 DAMs were identified. These included several key metabolites such as 4‐hydroxybenzoic acid, d‐glucose, argininosuccinic acid, salicylic acid‐2‐*O*‐glucoside, hesperetin‐7‐*O*‐glucoside and quercetin‐3‐*O*‐glucuronide. These DAMs showed significant positive or negative associations with several key DEGs, most notably *cytochrome P450 714C2‐like* (LOC107403538), *bidirectional sugar transporter SWEET15‐like* (LOC107404505) and *WRKY transcription factor 40* (LOC107406933), as illustrated in Figure 3C and detailed in Table S15. In the Fu_down_up versus T13_up_down group, 9 DAMs were identified, including allysine (6‐oxo‐dl‐norleucine), pyridoxal, 4‐*O*‐methylgallic acid, frangulanine, kaempferol‐3‐*O*‐(2″‐*O*‐acetyl)glucuronide, quercetin‐3‐*O*‐(6″‐*O*‐acetyl) glucoside, isolariciresinol‐9′‐*O*‐glucoside, kaempferol‐3‐*O*‐(6″‐malonyl) galactoside and quercetin‐3‐*O*‐(6″‐*O*‐*p*‐coumaroyl)sophoroside‐7‐*O*‐rhamnoside. These metabolites were significantly positively or negatively correlated with DEGs such as *WRKY transcription factor 71* (LOC107404587) and *transcription factor bHLH153‐like* (LOC107407179), as shown in Figure 3D and Table S15. Notably, WRKY transcription factors have been shown to play a significant role in regulating phytoplasma infection in jujube (Xue et al. 2019). The observed correlations between these WRKY transcription factors and key DAMs suggest that they may regulate the biosynthesis of specific secondary metabolites. Metabolites such as flavonoids, phenolic acids and sugars could play important roles in shaping the differential symptomatology observed between the susceptible genotype Fu and the tolerant genotype T13. These results provide important insights into the regulatory networks that potentially underlie phytoplasma tolerance in jujube.

### 2.5 The Expression Patterns of the WRKY Transcription Factors and Other Key Genes in Susceptible and Tolerant Jujube Genotypes Under Phytoplasma Infection

**FIGURE 4:**
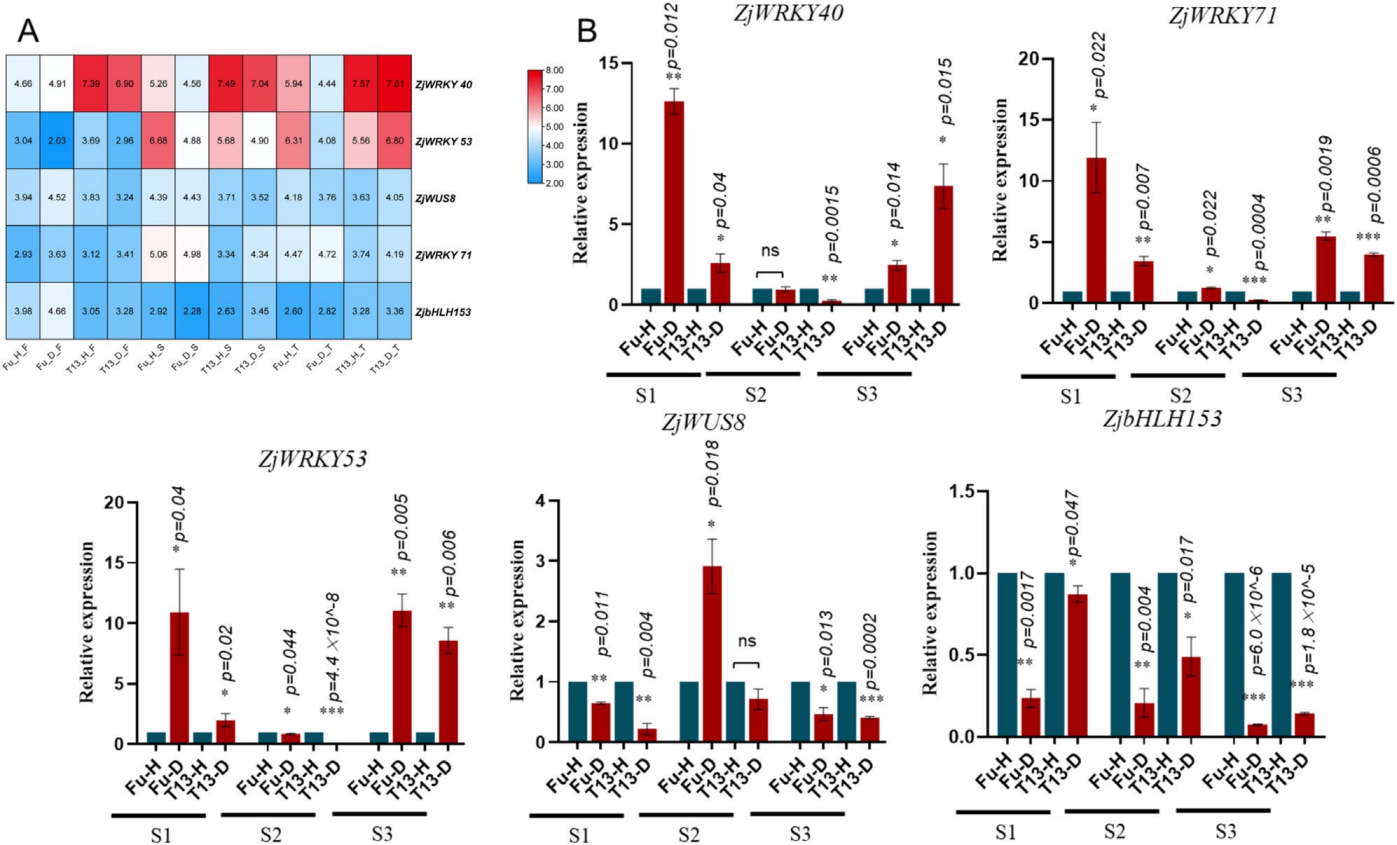
The expression patterns of *ZjWRKY40*, *ZjWRKY71*, *ZjWRKY53*, *ZjWUS8* and *ZjbHLH53* in Fu and T13 under phytoplasma infection at three growth stages (S1, S2 and S3) by RNA‐seq (A) and reverse transcription‐quantitative PCR analysis (B). The data in (A) are plotted as log_2_(transcripts per million [TPM]). *, ** and *** indicate a significant difference at *p* < 0.05, *p* < 0.01 and *p* < 0.001, respectively.

To validate the transcriptome findings, the expression patterns of key genes were examined using both transcriptome data and reverse transcription‐quantitative PCR (RT‐qPCR) analysis. As shown in Figure 4A (RNA‐seq data), the expression level of *ZjWRKY40* was significantly induced in T13, and its expression was consistently higher (at 2‐fold at both the S1 and S3 stages) than that in Fu. As shown in Figure 4B (RT‐qPCR data), the expression level of *ZjWRKY40* was significantly induced in T13 at the S1 and S3 stages, at 13‐fold and 7‐fold higher compared to that in Fu, respectively. In contrast, the expressions of *ZjWRKY71* and *ZjWRKY53* were significantly induced in Fu at the S3 stage (5‐fold and 10‐fold, respectively). However, in the RT‐qPCR analysis, the expression level of *ZjWRKY40* showed an increasing pattern in T13 from the S1 to S3 stages, while it was down‐regulated in Fu at the three growth stages (Figure 4B). The expression level of *ZjWRKY53* showed almost the same pattern. This was consistent with the expression patterns of the transcriptome data. Based on the RNA‐seq data, which showed that the differential expression of *ZjWRKY4* between Fu and T13 samples was more pronounced than that of *ZjWRKY53* and *ZjWRKY71*, *ZjWRKY40* was selected for subsequent functional characterisation to explore its potential central role in the phytoplasma response (Figure 4B).

### 2.6 The Function of *ZjWRKY40* in the Phytoplasma Tolerance of Jujube and Identification of *ZjWRKY40* Target Genes

**FIGURE 5:**
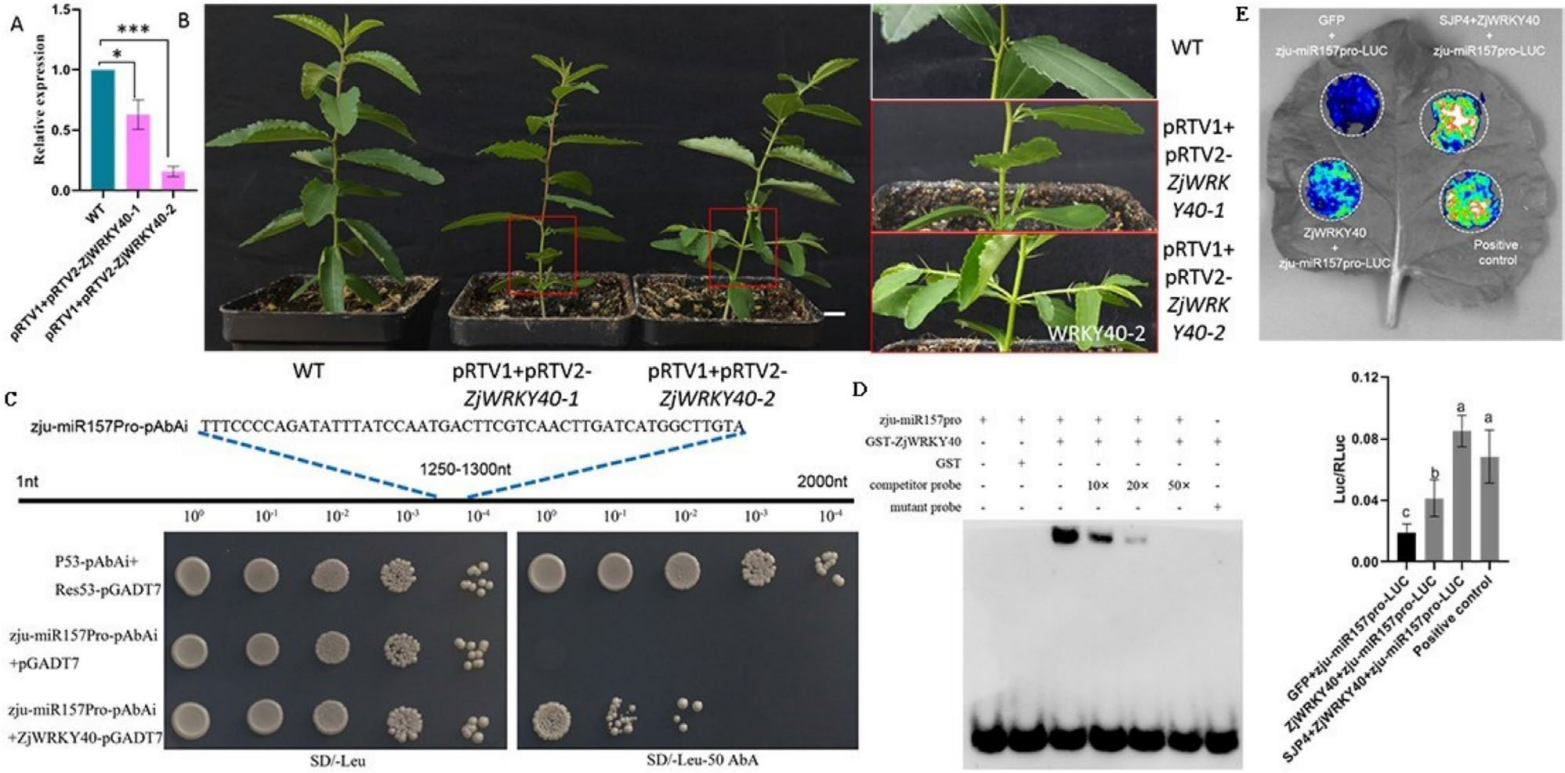
The function of *ZjWRKY40* in the phytoplasma tolerance and identification analysis of ZjWRKY40 target genes. The expression pattern of *ZjWRKY40* (A) and the phenotype of VIGS‐induced *ZjWRKY40* transiently silenced jujube plants (B). * and *** indicate a significant difference at *p* < 0.05 and *p* < 0.001, respectively. (C) The yeast one‐hybrid assay analysis between ZjWRKY40 and the promoter of *zju‐miR157*. (D) The electrophoretic mobility shift assay between ZjWRKY40 and the promoter of *zju‐miR157*. (E) The interaction analysis among SJP4_JWB_, ZjWRKY40, and the promoter of *zju‐miR157* by dual luciferase assay.

To investigate the functional role of *ZjWRKY40* in the phytoplasma tolerance, the virus‐induced gene silencing (VIGS)‐induced *ZjWRKY40* transient silencing assay was performed in jujube. As shown in Figure 5A, the expression of *ZjWRKY40* was significantly reduced in the VIGS‐silenced plants, confirming successful gene knockdown. Notably, these plants exhibited axillary bud germination, a typical symptom of JWB, which was absent in wild‐type plants (Figure 5B). This phenotypic difference indicates that *ZjWRKY40* positively correlates with the phytoplasma tolerance in jujube.

To further elucidate the molecular mechanism of *ZjWRKY40* in the regulation of phytoplasma tolerance, its target genes were identified. Previous study has shown that microRNAs play important roles in the plant response to phytoplasma infection (Wang, Luo, et al. 2024). Therefore, we examined the interaction between ZjWRKY40 and the promoter of *zju‐miR157*. In the yeast one‐hybrid (Y1H) assay, ZjWRKY40 was able to activate the reporter gene on SD/−Leu medium supplemented with 50 ng/mL aureobasidin A (AbA), whereas no growth was observed in the negative control, indicating direct binding of ZjWRKY40 to the *zju‐miR157* promoter (Figure 5C). This interaction was further confirmed by electrophoretic mobility shift assay, where ZjWRKY40 bound to the labelled probe of the *zju‐miR157* promoter, demonstrating its specific DNA‐binding capability (Figure 5D). To validate the regulatory role of *ZjWRKY40* in vivo, a dual‐luciferase assay was performed. Co‐expression of *35S::ZjWRKY40* and *zju‐miR157Pro‐LUC* in *Nicotiana benthamiana* leaves resulted in significantly increased luminescence compared to the GFP + *zju‐miR157Pro‐LUC* control (Figure 5E), confirming that ZjWRKY40 can activate *zju‐miR157* transcription. Moreover, co‐expression of *SJP4*_*JWB*_, *ZjWRKY40* and *zju‐miR157Pro‐LUC* further enhanced the luminescence signal compared to *ZjWRKY40* alone, suggesting that SJP4_JWB_ may interact with ZjWRKY40 to boost its transcriptional activation of *zju‐miR157* (Figure 5E). These findings provide strong evidence that ZjWRKY40 enhances the phytoplasma tolerance by directly targeting the promoter of *zju‐miR157* and that SJP4_JWB_ may serve as a co‐regulator to amplify this effect.

### 2.7 The Function Verification Analysis of *zju‐* *miR157*

**FIGURE 6:**
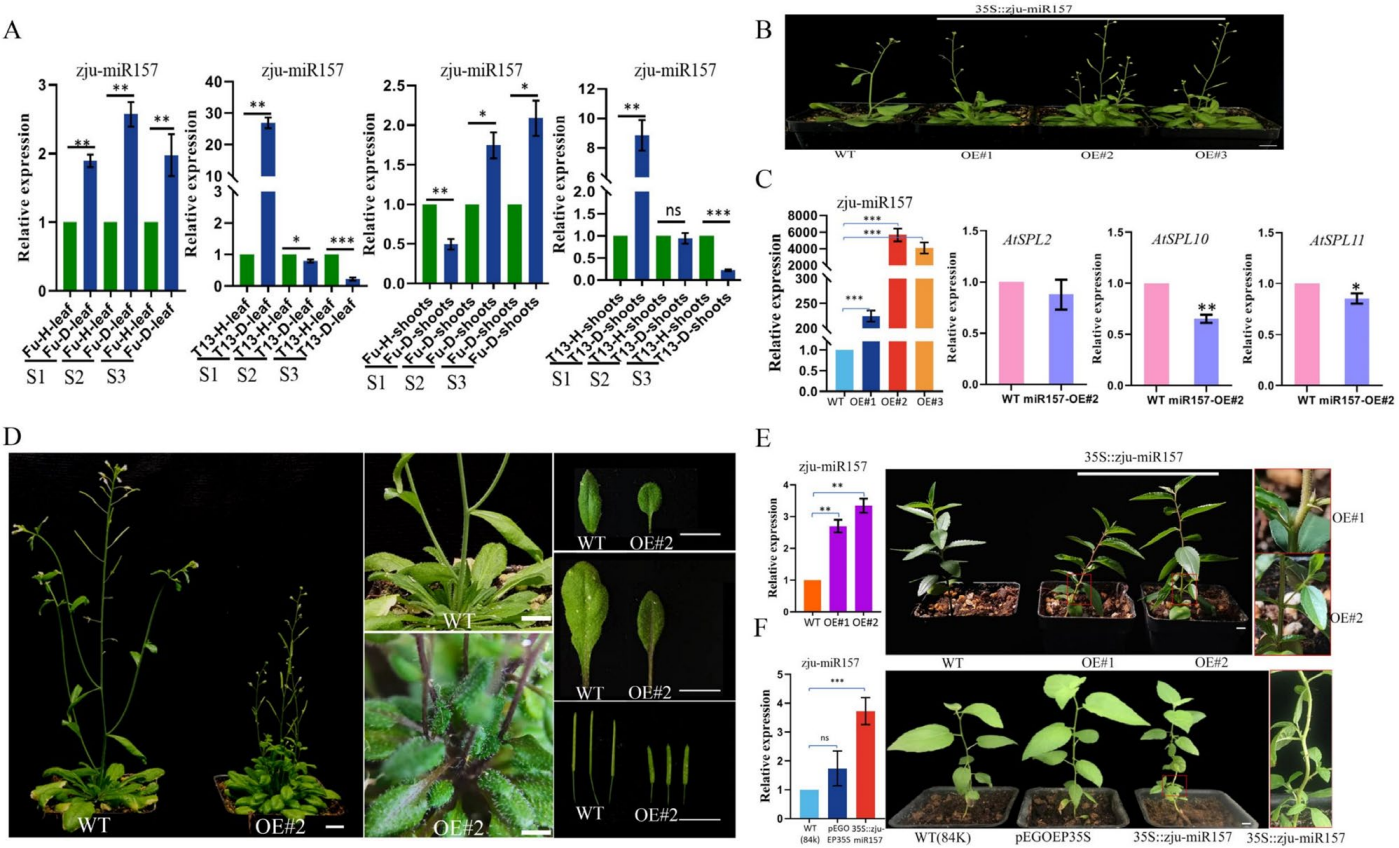
The expression patterns of *zju‐miR157* and the phenotype of plants overexpressing it ( *Arabidopsis thaliana* and poplar) and in transiently‐overexpressing jujube plants. (A) The expression pattern of *zju‐miR157* in the leaves and shoots of Fu and T13 under phytoplasma infection at three growth stages (S1–S3). (B) Representative images of *zju‐miR157*‐overexpressing *A. thaliana* seedlings at 25 days after being planted in soil. (C) The expression level of *zju‐miR157* and *AtSPLs* in three *zju‐miR157‐*overexpressing *A. thaliana* seedlings. (D) Representative images showing a morphological comparison between mature wild type (WT) and *zju‐miR157*‐overexpressing *A. thaliana* plants at 37 days after planting. Phenotypes presented include branching architecture, rosette leaves, mature leaves, and siliques. (E) The expression level of *zju‐miR157* in two jujube plants transiently overexpressing *zju‐miR157* and the phenotype observation. (F) The expression level of *zju‐miR157* in *zju‐miR157*‐overexpressing poplar plants and the phenotype observation. Bar = 1 cm. *, ** and *** indicate a significant difference at *p* < 0.05, *p* < 0.01 and *p* < 0.001, respectively.

To get insight into the functional role of z*ju‐miR157* in the phytoplasma tolerance, its expression pattern was first analysed in the leaves and shoots of Fu and T13 across the three developmental stages under phytoplasma infection. As shown in Figure 6A, the expression pattern of *zju‐miR157* was significantly induced in phytoplasma‐infected Fu. In contrast, in T13, expression was initially upregulated at the S1 stage, but notably decreased at S2 and S3 stages, coinciding with the recovery from infection. This dynamic expression pattern suggests that *zju‐miR157* may be involved in modulating jujube tolerance to phytoplasma, functioning differently in susceptible and tolerant genotypes. Moreover, the function of *zju‐miR157* was verified by transforming it into *A. thaliana* (Figure 6B), poplar (Figure 6F) and transiently overexpressing it in jujube (Figure 6E). The expression level of *zju‐miR157* in overexpressing *A. thaliana* (Figure 6C) and poplar plants (Figure 6F) was significantly higher than that in wild type. In addition, more shoot branches and rosette leaves, and smaller leaves were observed in *zju‐miR157*‐overexpressing *A. thaliana* plants compared to the wild type (Figure 6D). More axillary buds sprouted and grew in *zju‐miR157‐*overexpressing poplar plants (Figure 6F) and when transiently overexpressed in jujube plants (Figure 6E) compared to their wild type. Furthermore, the expression of *AtSPL10* and *AtSPL11* in *A. thaliana* decreased in *zju‐miR157‐*overexpressing plants (Figure 6C). All these results demonstrate that *zju‐miR157* negatively regulates jujube tolerance to phytoplasma by downregulating *SPL* genes.

### 2.8 The Function Verification Analysis of *ZjSPL3*

**FIGURE 7:**
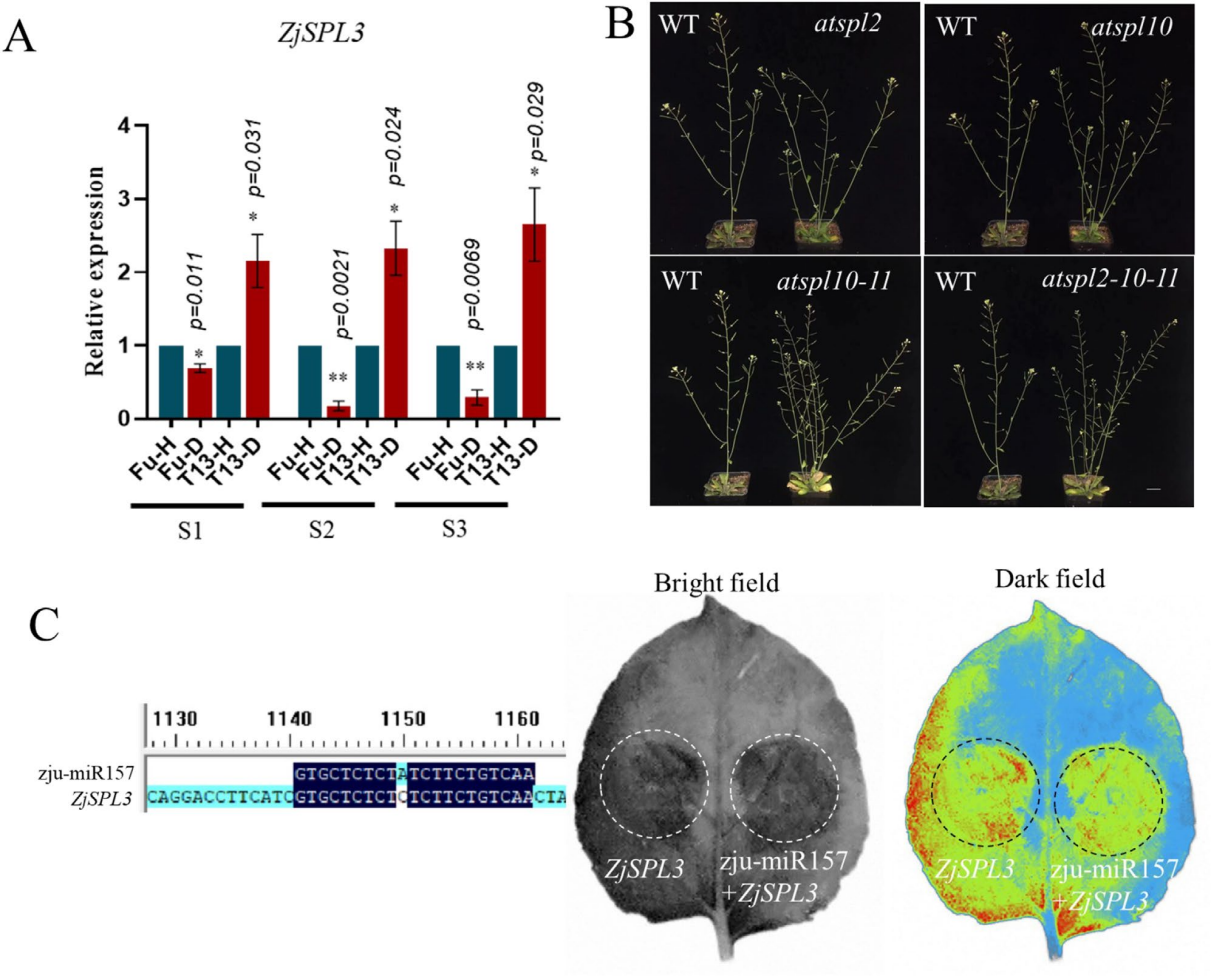
The expression patterns of *ZjSPL3* in Fu and T13 under phytoplasma infection at three growth stages (S1, S2 and S3) (A). The phenotype observation of *ZjSPL3* homologue mutants in *Arabidopsis thaliana* (*AtSPL2/10/11*) (B). The sequence complementation and luciferase complementation assay analysis between *zju‐miR157* and *ZjSPL3* (C). * and ** indicate a significant difference at *p* < 0.05 and *p* < 0.01, respectively.

As *ZjSPL3* is the main target of *zju‐miRNAs* that regulate the witches' broom symptom in jujube (Wang, Bai, et al. 2024; Wang, Luo, et al. 2024), the expression level of *ZjSPL3* in Fu and T13 under phytoplasma infection at the three growth stages was examined. As shown in Figure 7A, the expression level of *ZjSPL3* was significantly upregulated in T13 across all stages, while it was downregulated in Fu. This trend suggests that higher *ZjSPL3* expression may contribute to the phytoplasma tolerance observed in T13. In addition, the phenotype observation of *ZjSPL3* homologue mutants in *A. thaliana* (*AtSPL2/10/11*) showed more shoot branches (Figure 7B), which further confirms that *ZjSPL3* is positively correlated with the phytoplasma tolerance. The target gene of zju‐miR157 was found to be *ZjSPL3* by sequencing comparison analysis, and luciferase complementation assay confirmed that zju‐miR157 could target *ZjSPL3*, although the luminescence signal was relatively weak (Figure 7C), which might indicate that zju‐miR157 could regulate the expression of *ZjSPL3* to confirm the phytoplasma tolerance in T13.

### 2.9 Functional Verification of SJP4_JWB_ and Its Interaction With ZjWRKY40

**FIGURE 8:**
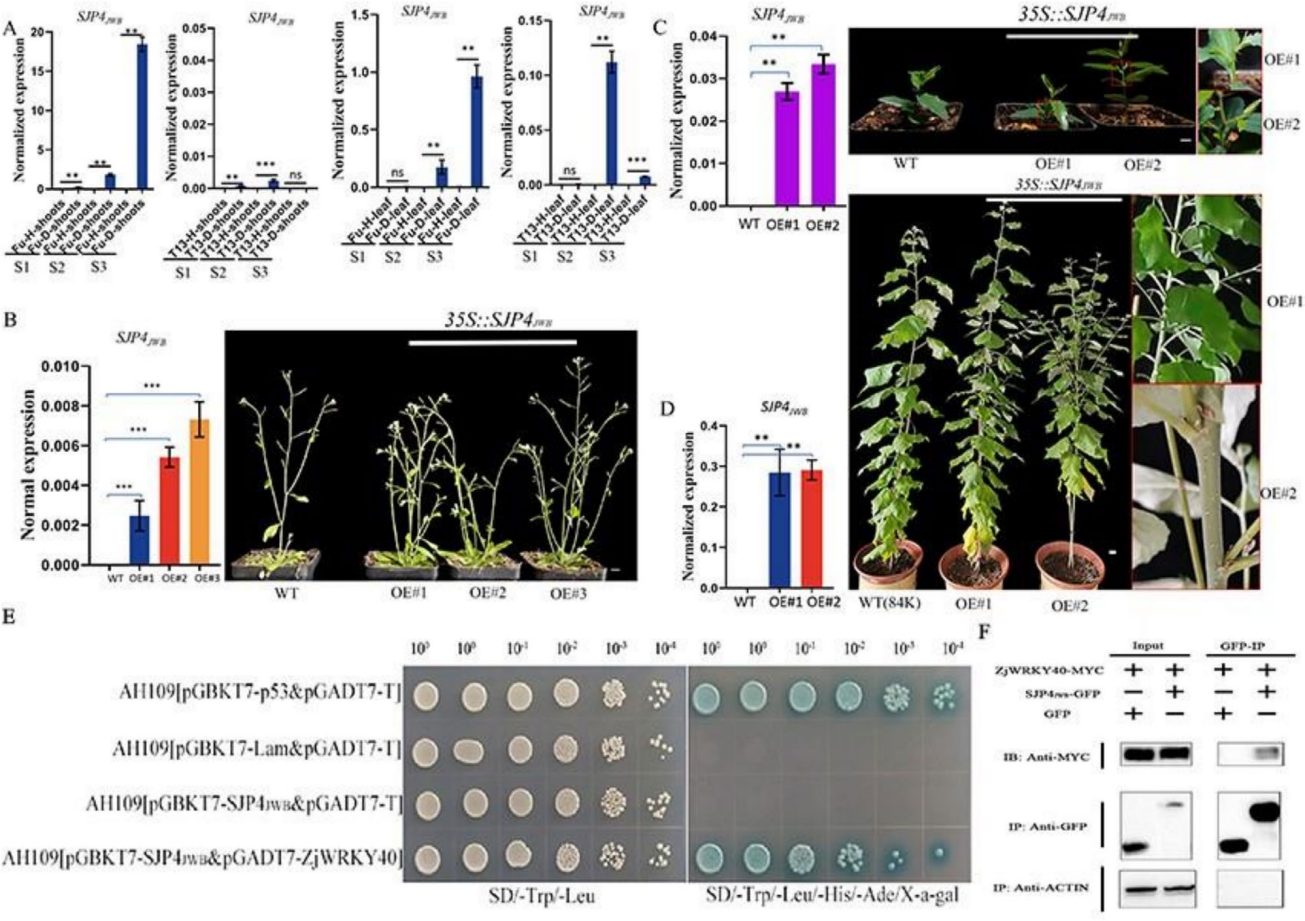
The function verification analysis of SJP4_JWB_ and interaction analysis between SJP4_JWB_ and ZjWRKY40. (A) The expression pattern of *SJP4*_*JWB*_ in the leaves and shoots of Fu and T13 under phytoplasma infection at three growth stages (S1–S3). (B) The expression level of *SJP4*_*JWB*_ in overexpressing *Arabidopsis thaliana* plants and the phenotype observation. (C) The expression level of *SJP4*_*JWB*_ in transiently overexpressing sour jujube plants and the phenotype observation. (D) The expression level of *SJP4*_*JWB*_ in overexpressing poplar plants and the phenotype observation. Yeast two‐hybrid (E) and co‐immunoprecipitation (F) analysis between SJP4_JWB_ and ZjWRKY40. Bar = 1 cm. ** and *** indicate a significant difference at *p* < 0.01 and *p* < 0.001, respectively.

Using comparative genome analysis, a novel phytoplasma effector protein was identified and designated SJP4_JWB_ (Figure S8). To determine its functional role, the expression level of *SJP4*_*JWB*_ was analysed in Fu and T13 under phytoplasma infection at the three developmental stages. As shown in Figure 8A, *SJP4*_*JWB*_ expression was strongly induced in Fu from S1 to S3 in both leaves and shoots, while only slight induction was observed in T13 at the S2 stage. Overall, expression levels were significantly higher in the susceptible genotype, suggesting a role in promoting disease symptoms. To validate its function, *SJP4*_*JWB*_ was overexpressed into *A. thaliana* , poplar, and transiently in jujube. The expression level of *SJP4*_*JWB*_ was significantly higher in overexpressing *A. thaliana* and poplar plants compared to wild‐type controls. Similarly, its expression was markedly induced in overexpressing jujube plants (Figure 8B–D). In addition, compared to the wild type, the three *35S::SJP4*_*JWB*_ *A. thaliana* overexpression lines (OE#1, OE#2 and OE#3) exhibited increased shoot and branch formation, resembling the witches' broom symptom. Similarly, in *35S::SJP4*_*JWB*_ poplar plants (OE#1 and OE#2), more axillary buds developed compared to the wild type (84K) (Figure 8B,D). Moreover, the more axillary bud growth phenotypes were observed in jujube plants transiently overexpressing *SJP4*_*JWB*_ (Figure 8C). These results demonstrated that SJP4_JWB_ could promote the shoot and axillary bud growth as well as witches' broom symptoms in jujube.

Because SJP4_JWB_ could induce the phytoplasma disease symptom in plants, it was hypothesised that SJP4_JWB_ might interact with ZjWRKY40 to regulate the jujube phytoplasma tolerance. Thus, the interaction between ZjWRKY40 and SJP4_JWB_ was tested. As shown in Figure 8E,F, yeast two‐hybrid and co‐immunoprecipitation assays demonstrated that *S*JP4_JWB_ interacted with ZjWRKY40.

## 3 Discussion

### 3.1 Identification of Jujube Phytoplasma‐Tolerant Genotype

When a jujube tree is infected by phytoplasma, the diseased trees are normally removed; if the situation in the orchard is severe, the clear orchard method is conducted, which causes great economic loss (Wang, Luo, et al. 2024). To prevent phytoplasma disease, other several control methods have been applied, such as chemical and biological treatments or effective cultivation management. For example, in jujube, high activities of glutathione S‐transferase and oxytetracycline hydrochloride treatment can somewhat prevent phytoplasma symptomatology (Xue et al. 2020; Yang, Shen, et al. 2023). Chemical or biological controls are widely conducted on other plants in response to phytoplasma infection. For example, exogenous salicylic acid and benzothiadiazole induce systemic acquired resistance in sugarcane against phytoplasma‐induced white leaf disease (Ratchaseema et al. 2021). In *Camptotheca acuminata* , the accumulation of isochlorogenic acid B, atractylenolide II and 3‐methoxybenzoic acid has been identified as potential defence metabolites against witches' broom (Qiao et al. 2023). The endoplasmic reticulum plays a vital role in producing immune‐related proteins and signalling components during plant–phytoplasma interactions (Inaba et al. 2023; Musetti et al. 2023). However, a major limitation of these methods is their frequent inefficacy against severe infections, coupled with the phytotoxic damage they often inflict on the host plants. Thus, a more effective method to prevent phytoplasma disease is to identify and select resistant or tolerant genotypes, which can significantly reduce chemical pesticide usage and lower production costs, aligning with the principles of green plant protection and ecological agriculture. In this study, two genotypes were comprehensively evaluated: Fu (phytoplasma‐susceptible) and T13 (tolerant). Through detailed phenotypic observations and analysis of the expression level of *ZjTMK*, the differential phytoplasma tolerance of these genotypes was confirmed, consistent with previous findings (Wang, Liu, et al. 2022). These results highlight the potential of T13 as a valuable resource for uncovering the molecular mechanisms underlying the phytoplasma tolerance, which could inform breeding strategies aimed at improving jujube resilience to JWB. However, it is important to note that the tolerance mechanism studied herein implies the host's ability to maintain growth despite pathogen presence, which differs from resistance that restricts pathogen accumulation. From an epidemiological perspective, tolerant plants, while agronomically desirable for yield, could potentially serve as asymptomatic reservoirs for the phytoplasma, facilitating its spread by insect vectors. Therefore, the long‐term strategic goal for sustainable disease management should be to translate insights from tolerance mechanisms into the development of resistant genotypes that effectively reduce pathogen titre and interrupt the transmission cycle.

### 3.2 *ZjWRKY40* Functions Importantly in the Phytoplasma Tolerance

Despite advances in characterising phytoplasma effectors (e.g., SAP05 and SAP11) that induce severe symptoms (Oshima et al. 2023), little is known about how plants resist infection, a knowledge gap exacerbated by the limited availability of tolerant germplasm. While studies on recovered grapevine have linked resistance to hormonal and metabolic reprogramming, this gap underscores the value of multi‐omics (Pagliarani et al. 2020). For example, transcriptomic analysis in jujube has highlighted the role of abscisic acid signalling, demonstrating the power of integrated approaches to unravel complex tolerance mechanisms (Ma et al. 2023). Comparative transcriptome analysis between JWB‐tolerant Indian jujube Cuimi and susceptible jujube Huping showed that protein ubiquitination, cell wall biogenesis, transcription factor activity might function importantly in their differential response to phytoplasma infection (Xu et al. 2023). In addition, trunk injection of oxytetracycline hydrochloride has been shown to reverse JWB symptoms in jujube by modulating pathways related to signalling, photosynthesis, plant hormone metabolism and transduction (Yang, Shen, et al. 2023). In cucumber, phytoplasma‐like symptoms were associated with increased levels of auxin, salicylic acid and jasmonic acid, while transcriptome data suggested that hormone signalling, phenylpropanoid biosynthesis and phenylalanine metabolism pathways contribute to disease symptom development (Wang, Hu, et al. 2022). In the current study, integrated transcriptomics and metabolomics analyses was employed to elucidate the underlying molecular mechanism of the phytoplasma tolerance in jujube. In the Fu_down_down versus T13_up_up group, a total of 47 DAMs were identified, which might be correlated to *WRKY transcription factor 40* (LOC107406933). Notably, the expression of key regulatory genes like *ZjWRKY40* was strongly upregulated in T13 under phytoplasma infection. This transcriptional reprogramming might be directly correlated with the metabolomic profiles, such as the antimicrobial and antioxidant metabolites. These compounds may then directly inhibit phytoplasma multiplication or reinforce sieve element defences, thereby constituting a biochemical basis for the observed tolerance phenotype. In addition, VIGS of *ZjWRKY40* in jujube confirmed that *ZjWRKY40* is positively correlated with the phytoplasma tolerance. WRKYs have been widely studied in different plants and play an important role in various plant disease tolerance (Saha et al. 2023; Liu et al. 2024). In wheat, overexpression of *TaWRKY74* enhanced its resistance to ‘*Ca*. P. tritici’, and further study found that the effector SWP12 could induce phytoplasma colonisation in wheat leaves by degrading *TaWRKY74* (Bai et al. 2022). Notably, *WRKY transcription factor 40* has been shown to play a significant role in regulating phytoplasma infection in jujube (Xue et al. 2019). Thus, *ZjWRKY40* could be the key gene regulating the main DAMs to regulate the phytoplasma tolerance in jujube.

### 3.3 *ZjWRKY40‐zju‐miR157* Module Contributes to the Phytoplasma Tolerance

In plants, phytoplasma effectors can degrade their target transcription factors to modulate host immunity; in particular, the TCP transcription factors are the most common targets (Correa Marrero et al. 2024). For example, SJP1/2 suppress the expression of *miR319f_1*, leading to the accumulation of *ZjTCP2* at both post‐transcriptional and protein levels, which ultimately contributes to the leaf shrinkage under phytoplasma infection in jujube (Ma, Zheng, et al. 2024). Beyond TCP transcription factors, SJP1_JWB_ has been shown to interact with *ZjERF18* transcription factor to regulate the process of phytoplasma infection (Wang, Luo, et al. 2024). In wheat, the effector SWP12 targets and destabilises *TaWRKY74*, increasing susceptibility to ‘*Ca*. P. tritici’ infection (Bai et al. 2022). These findings collectively highlight that phytoplasma effectors regulate multiple transcription factors to mediate host responses.

**FIGURE 9:**
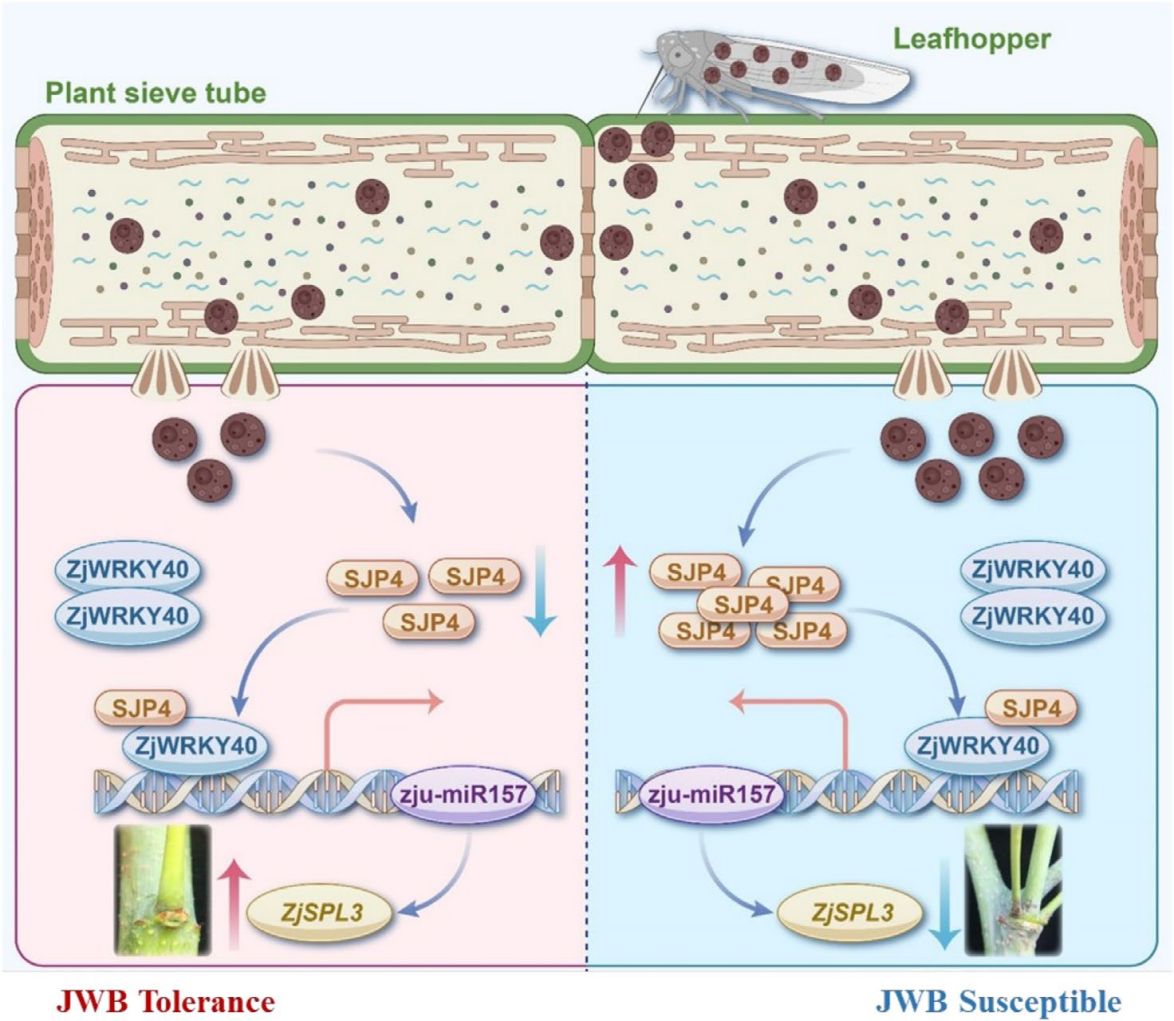
The model of SJP4_JWB_‐ZjWRKY40‐zju‐miR157 module regulated the differential phytoplasma tolerance between phytoplasma‐susceptible genotype Fu and phytoplasma‐tolerant genotype T13.

Additionally, miRNAs have been demonstrated to play a crucial role under phytoplasma infection (Wang, Luo, et al. 2024; Shao et al. 2016). To date, three SJP_JWB_ effectors, SJP1, SJP2 and SJP3, have been identified in jujube phytoplasma infection (Ma, Huang, et al. 2024; Ma, Zheng, et al. 2024; Deng et al. 2024). These effectors play critical roles in the manipulation of lateral bud germination, leaf shrinkage, shoot branching and pistil reversion. In the current study, a new effector was identified from the SJP family. It was designated as SJP4_JWB_ and could interact with ZjWRKY40. In addition, ZjWRKY40 directly targets the promoter of *zju‐miR157*, and *zju‐miR157* regulates the phytoplasma tolerance in jujube. Based on these findings, it is proposed a regulatory model explaining how the SJP4_JWB_ *–ZjWRKY40–zju‐miR157* module contributes to the phytoplasma tolerance (Figure 9). Concisely, in phytoplasma susceptible genotype Fu, the expression level of *ZjWRKY40* was down‐regulated; however, the SJP4_JWB_ effector accumulated more with the development of severe JWB symptoms. Elevated levels of SJP4_JWB_, together with *ZjWRKY40*, enhance the transcription of *zju‐miR157*. The high expression of *zju‐miR157* prevented the expression of *ZjSPL3*, resulting in the formation of JWB symptoms. In contrast, in the phytoplasma‐tolerant genotype T13, *ZjWRKY40* expression is significantly upregulated during phytoplasma infection, while *SJP4*_*JWB*_ expression decreases during the recovery from JWB symptoms. The lower accumulation of SJP4_JWB_, in combination with elevated *ZjWRKY40*, results in reduced induction of *zju‐miR157*, which relieves its repression on *ZjSPL3*. Increased expression of *ZjSPL3* contributes to tolerance against JWB symptoms in T13. However, several questions need to be explored: SJP4_JWB_ is less abundant in tolerant T13 compared to Fu, which might be due to the activated tolerant metabolites in T13 preventing the multiplication of the phytoplasma that finally secrete less effectors. The other one is that the metabolic changes regulated by the SJP4_JWB_ *–ZjWRKY40–zju‐miR157* module remain to be fully explored. In addition, the phytoplasma effector could target some transcription factors through destabilising them to induce the phytoplasma symptom (Bai et al. 2022; Correa Marrero et al. 2024). Thus, another question is whether SJP4_JWB_ degrades or stabilises ZjWRKY40 to confer the phytoplasma tolerance in T13. Future studies should focus on these questions and identify the key metabolites modulated by this regulatory pathway, which may be applied to manage phytoplasma disease spread in jujube orchards.

In conclusion, phenotype observation and phytoplasma testing demonstrated that Fu is a phytoplasma‐susceptible genotype, whereas T13 exhibits tolerance to phytoplasma. Integrated transcriptomic and metabolomic correlation analyses revealed that *ZjWRKY40* plays a pivotal role in the phytoplasma tolerance. Moreover, ZjWRKY40 directly binds to the promoter of *zju‐miR157*, a microRNA that negatively regulates the phytoplasma tolerance by repressing the expression of *ZjSPL3*, a key downstream target. Importantly, a novel phytoplasma effector, designated SJP4_JWB_, was identified. This effector interacts with ZjWRKY40 and enhances its transcriptional activity, ultimately contributing to the regulation of *zju‐miR157* and the jujube defence response. Together, these findings uncover a novel regulatory pathway, the SJP4_JWB_ *–ZjWRKY40–zju‐miR157* axis, that plays a critical role in modulating the phytoplasma tolerance in jujube, offering potential targets for breeding and disease control strategies.

## 4 Experimental Procedures

### 4.1 Plant Materials

The phytoplasma susceptible genotype Fu and the tolerant genotype T13 were cultivated at in Fuping experimental facility of jujube, Hebei Agriculture University. Scions from both genotypes were grafted onto phytoplasma‐infected Fu rootstocks in May, and the samples were collected from three growth stages, denoted as Fu (First_S1, Second_S2 and Third_S3) and T13 (First_S1, Second_S2 and Third_S3). In the transcriptome and metabolome analysis, the three growth stages were denoted as Fu (First_F, Second_S and Third_T) and T13 (First_F, Second_S and Third_T), following the protocol described by Wang, Hu, et al. (2022); Wang, Liu, et al. (2022). Among them, S1 (F), S2 (S) and S3 (T) represented 1 month, one and a half months, and 2 months after grafting, respectively. Scions grafted onto healthy rootstocks that were not infected by phytoplasma were used as negative controls. The observation of the symptoms of both genotypes was performed for 2 years after grafting, and the observation confirmed T13 as tolerant and Fu as a susceptible genotype. After phenotype observation, the samples were rapidly frozen in liquid nitrogen and stored in −80°C for subsequent analyses, including phytoplasma content detection (based on *ZjTMK* expression) (Xue et al. 2020), transcriptomic and metabolomic analyses.

Seeds of the Columbia ecotype of *A. thaliana* were surface‐sterilised using a sodium hypochlorite solution. They were then kept in the dark at 4°C for 2 days to allow stratification. Afterward, the seeds were sown onto Murashige and Skoog (MS) medium (½ strength MS, 0.22 g/100 mL) supplemented with 0.8 g/100 mL agar and 1 g/100 mL sucrose (Wang, Luo, et al. 2024). After germination, seedlings were transferred to small pots with 6 cm width and length and grown under controlled environmental conditions. Sour jujube seedlings were cultivated under conditions of 25°C–28°C with a 16‐h light. 84K poplar (*Populus alba* × *Populus glandulosa*) was grown at approximately 25°C with an average photoperiod of 16‐h light. *Nicotiana benthamiana* plants were grown in pots with an average daily temperature of 22°C–24°C under 16 h of light.

### 4.2 Transcriptome Sequencing Analysis

After RNA extraction, reverse transcription, library construction and sequencing were performed by Shanghai Majorbio Bio‐pharm Biotechnology Co. Ltd. (Shanghai, China) following the manufacturer's (Illumina) protocols. This process referred to the methods described by Wang, Hu, et al. (2022); Wang, Liu, et al. (2022). The fastp software was used for reads quality control, the HISAT2 software was applied to align the clean reads to the jujube reference genome (Chen et al. 2018; Kim et al. 2015). The mapped reads including multiple mapped and uniquely mapped were selected to do the annotation and DESeq2 was conducted for the differential expression analysis with uniquely mapped reads, and genes with log_2_FC ≧ |1| and false discovery rate (FDR)‐adjusted *p*‐values < 0.05 (DESeq2) were significant DEGs (Love et al. 2014). GO functional enrichment, especially the biological process (BP) term, and KEGG pathway analysis were carried out by Goatools (v. 0.6.5; https://github.com/tanghaibao/GOatools) and Python scipy software (v. 1.0.0), respectively. Meanwhile, to mitigate bias caused by large variations in gene counts among GO terms, we set a minimum gene count threshold for each term. Only GO terms containing at least 5 genes were included in subsequent analysis. This step filters out overly specific terms with insufficient statistical power. To ensure the overall statistical rigour of the analysis and avoid false positive results, we applied the Benjamini–Hochberg (BH) method to adjust the *p*‐values obtained from the hypergeometric test for FDR. Finally, GO terms with an adjusted *p*‐value (FDR) < 0.05 were defined as significantly enriched in the differential expression gene set.

### 4.3 Widely Targeted Metabolome Analysis

After sample grinding, metabolites were extracted with a 70% methanol solution, followed by filtration. The resulting extracts were analysed using a UPLC‐ESI‐MS/MS system. The analysis was performed with a SHIMADZU Nexera X2 UPLC and an Applied Biosystems 4500 Q TRAP mass spectrometer. All analyses were conducted by Metware (Wuhan, China). Analytical conditions were as follows. The effluent was introduced into an electrospray ionisation (ESI) source connected to a triple quadrupole‐linear ion trap (QTRAP‐MS). Instrument tuning and mass calibration were performed using 10 μM and 100 μM polypropylene glycol solutions in triple quadrupole (QQQ) and LIT modes, respectively. Multiple reaction monitoring (MRM) transitions specific to each metabolite were monitored according to their retention time windows. Differentially accummulated metabolites (DAMs) between comparisons were identified based on the criteria of VIP (variable importance in projection) ≥ 1 and absolute log_2_FC ≥ 1. VIP scores were obtained from an orthogonal partial least squares‐discriminant analysis (OPLS‐DA) model. To validate the model and avoid overfitting, a permutation test with 200 iterations was performed. The annotation of metabolites was performed based on KEGG compound and HMDB database.

### 4.4 Expression Analysis of Some Key Genes

Plant RNA Extraction Kit (Tiangen Biotech) was used for total RNA extraction. The first‐strand cDNA was synthesised from 500 ng of total RNA using the cDNA First‐Strand Synthesis Premix (Tiangen Biotech). miRNA was extracted using the miRcute Polyphenol Polysaccharide Plant miRNA Extraction Kit (Tiangen Biotech), and then reverse transcribed into the first‐strand miRNA cDNA using the miRcute Enhanced miRNA cDNA First‐Strand Synthesis Kit (Tiangen Biotech). The quality and concentration of DNA, RNA, and miRNA were further evaluated using NanoDrop2000. All DNA, cDNA and miRNA cDNA samples were stored at −20°C for next application.

Primers were designed and synthesised based on the mature sequence of zju‐miR157, as well as for SJP4_JWB_, *TMK*_*JWB*_, and *ZjWRKY40* (Table S1). Using the cDNA and miRNA cDNA of Fu and T13 three growth stages as templates, RT‐qPCR was performed to detect the expression of *TMK*_*JWB*_, *SJP4*_*JWB*_ and zju‐miR157. RT‐qPCR was also used to detect the expression levels of *zju‐miR157* and *SJP4*_*JWB*_ in their overexpressing *A. thaliana* , poplar, transiently overexpressing jujube plants and wild‐type plants.

For details, 20 μL reaction mixture contained 10 μL of 2 × SuperReal PreMix Plus (Tiangen Biotech), 0.4 μL of each 10 μM primer, 1 μL of cDNA and 8.2 μL of double‐distilled water (ddH_2_O). The thermal programme consisted of pre‐incubation at 95°C for 15 min, followed by 40 cycles of 95°C for 10 s, 55°C–63°C for 20 s and 72°C for 20 s. *Actin* was used as the housekeeping gene. When using miRNA cDNA as the template, a three‐step method was adopted. The 20 μL reaction mixture contained 10 μL of 2 × miRcute Plus miRNA Premix (Tiangen Biotech), 0.4 μL of each 10 μM primer, 1 μL of miRNA cDNA and 8.2 μL of ddH_2_O. The thermal programme consisted of pre‐incubation at 95°C for 15 min; followed by 5 cycles of 94°C for 20 s, 63°C–65°C for 30 s and 72°C for 34 s; and then 40 cycles of 94°C for 20 s and 60°C for 34 s. *U6* was used as the housekeeping gene (Table S1). Relative expression levels and normalised expression levels were calculated using the 2^−ΔΔCt^ method, which assumes optimal and equal amplification efficiency (*E* = 2.0) for all primer pairs. Future studies incorporating efficiency‐corrected models (e.g., the Pfaffl method) could provide an additional layer of quantitative precision.

### 4.5 Functional Verification of *ZjWRKY40*

Virus‐induced gene silencing (VIGS) of *ZjWRKY40* in sour jujube was conducted following the method described by Wang et al. (2025). Briefly, the recombinant plasmid pTRV2 *‐ZjWRKY40* was transferred into *Agrobacterium tumefaciens* GV3101, and a mixture of pTRV1 and pTRV2*‐ZjWRKY40* (1:1 ratio) was infiltrated into the cotyledons of sour jujube seedlings. A second infiltration was performed after 7 days of infection. Seven to 10 days following the second injection, leaf samples were collected for RNA extraction and quantification of *ZjWRKY40* expression to confirm gene silencing in the VIGS‐treated plants. Phenotypic observations were carried out 7 days post‐infiltration to assess the effect of the *ZjWRKY40* silencing, using both VIGS‐induced and wild‐type sour jujube plants for comparison.

### 4.6 The Interaction Analysis Between ZjWRKY40 and the Promoter of *zju‐miR15*7

#### 4.6.1 Electrophoretic Mobility Shift Assay

A 5′‐biotinylated oligonucleotide (5′‐ATGCATGCATGCATGCATGCATGC‐3′) was used to assess protein–DNA binding activity. The probe was incubated with the nuclear protein extracts at room temperature for 30 min. The reaction mixture was then electrophoresed on a non‐denaturing 6% polyacrylamide gel in 0.5 × TBE buffer for 1 h. Following electrophoresis, the gel was transferred onto Biodyne B nylon membranes (Pall Corporation). Signal detection was performed using a chemiluminescent elecytrophoretic mobilty shift assay (EMSA) kit according to the manufacturer's instructions, and visualisation was carried out using the ChemiDoc XRS system (Bio‐Rad).

#### 4.6.2 Yeast One‐Hybrid Assay

To determine whether ZjWRKY40 binds to promoter zju‐miR157, a yeast one‐hybrid (Y1H) assay was performed. The predicted binding region of the *zju‐miR157* promoter was cloned into the pAbAi vector to create the bait construct (zju‐miR157pro‐pAbAi). The *ZjWRKY40* coding sequence was inserted into the pGADT7 vector to generate the prey construct (pGADT7‐WRKY40). The bait vector and the positive control plasmid (p53‐pAbAi) were linearised using the restriction enzyme BbsI, following standard digestion protocols. The linearised DNA fragments were purified and transformed into Y1H Gold yeast strains, which were then plated onto appropriate SD/−Ura selective medium and incubated at 30°C for 4–5 days. After transformation, aureobasidin A (AbA) sensitivity screening was conducted to determine the minimal inhibitory concentration of AbA required for selection. Competent cells of zju‐miR157pro‐pAbAi and p53‐pAbAi yeast strains were prepared. The prey plasmids (pGADT7‐WRKY40 and empty pGADT7) were then transformed into the bait‐containing competent cells. Transformed yeast cells were plated onto SD/−Leu/−Ura (SD−UL) medium with and without AbA. Plates were incubated at 30°C for 4–5 days, and interaction was assessed based on yeast growth in the presence of AbA.

### 4.7 Transcriptional Analysis of Zju‐miR157 Regulated by ZjWRKY40 and SJP4_JWB_

Constructs containing *GFP+zju‐miR157pro‐LUC*, *ZjWRKY40+zju‐miR157pro‐LUC*, *SJP4*_*JWB*_ *+ZjWRKY40+zju‐miR157Pro‐LUC* and positive control (*D‐luciferin*) vectors were generated. Fully expanded leaves of *N. benthamiana* were selected for *Agrobacterium*‐mediated infiltration using a 1 mL syringe (without the needle) from the abaxial (underside) surface of the leaves. To ensure experimental consistency, control and test constructs were co‐infiltrated into different regions of the same leaf. This approach minimised background variation caused by developmental or environmental factors. At 48–72 h post‐injection, approximately 100 mg of treated leaf tissue was collected and ground into a fine powder using liquid nitrogen. 1 mL of pre‐cooled lysis buffer was added, followed by vortexing for thorough mixing. Samples were lysed on ice for 15 min, then centrifuged at 12,000 *g* for 5 min at 4°C. The resulting supernatant was collected for luciferase activity measurement. 20 μL of the lysate was added to a black 96‐well plate, followed by 100 μL of luciferase substrate working solution. Luminescence was immediately detected using a chemiluminescence reader and recorded as L_1_. Next, 100 μL of *Renilla* substrate working solution was added to the same well, and the luminescence was again measured and recorded as L_2_. Finally, to determine promoter activity, the relative luminescence ratio was calculated as Luc/RLuc = L_1_/L_2_. Expression differences among the treatment groups were then evaluated after normalisation to the internal control.

### 4.8 Functional Analysis of Zju‐miR157 and SJP4_JWB_

The *zju‐miR157* and *SJP4*_*JWB*_ were cloned into the overexpression vector pEGOEP35S‐H‐ *green fluorescent protein* (GFP) and transformed into *A. tumefaciens* GV3101. The flower dip method was used to transform wild‐type *A. thaliana* , and hygromycin‐containing medium was used to screen for positive seedlings. The expression levels of *zju‐miR157* and *SJP4_JWB_* were confirmed by RT‐qPCR, and stable overexpression lines were obtained. Phenotypic changes, such as global evaluation of aerial parts, in the transgenic plants were then observed and recorded.

The 35S::zju‐miR157‐GFP and 35S::SJP4_JWB_‐GFP constructs were individually introduced into *A. tumefaciens* GV3101. These constructs were then injected into the cotyledons of sour jujube seedlings to achieve transient transformation. The wild‐type sour jujube seedlings were used as controls. The phenotype of axillary bud germination was observed, and the expression level of *zju‐miR157* and *SJP4*_*JWB*_ was measured 30 days after injection. *35S::zju‐miR157‐GFP* and *35S::SJP4*_*JWB*_ *‐GFP* were transformed into 84K poplar using the leaf disc method, resulting in the generation of transgenic poplar plants. Then the phenotypic changes, such as global evaluation of aerial parts, were observed.

### 4.9 The Interaction Analysis Between SJP4_JWB_ and ZjWRKY40

#### 4.9.1 Yeast Two‐Hybrid Assay

To investigate the interaction between SJP4_JWB_ and ZjWRKY40, the coding sequence of SJP4_JWB_ was cloned into the pGBKT7 vector to generate the bait construct (pGBKT7‐SJP4_JWB_) for yeast two‐hybrid (Y2H) analysis. Before the interaction assay, autoactivation testing was performed by transforming pGBKT7‐SJP4_JWB_ into the Y2H gold yeast strain and plating the transformants on SD/−Trp/−Leu medium. Colonies were then transferred to SD/−Trp/−Leu/−His and SD/−Trp/−Leu/−His/−Ade plates to verify the absence of autoactivation. For the interaction assay, pGBKT7‐SJP4_JWB_ (bait), pGBKT7‐p53 (positive control) and *pGADT7‐ZjWRKY40* (prey) were co‐transformed into the Y2H Gold yeast strain. Co‐transformants were initially selected on SD/−Trp/−Leu medium and subsequently transferred to higher‐stringency media (SD/−Trp/−Leu/−His and SD/−Trp/−Leu/−His/−Ade). Interaction was inferred from yeast growth on these selective plates.

#### 4.9.2 Co‐Immunoprecipitation Assay

Co‐immunoprecipitation (Co‐IP) assay was performed as described in Wang et al. (2025), with slight modification. Briefly, the *35S::SJP4*_*JWB*_ *‐GFP* and *35S::ZjWRKY40‐FLAG* vectors were transformed into *A. tumefaciens* GV3101 and infiltrated into 1‐month‐old *N. benthamiana* plants. After 48 h, infiltrated leaves were harvested and immediately frozen in liquid nitrogen for total protein extraction. The Co‐IP procedure included bead preparation, antibody binding, bait protein capture, denaturation by boiling, SDS‐PAGE electrophoresis, protein transfer to membranes and antibody hybridisation. Protein–protein interactions were detected by chemiluminescence, and images were visualised and captured using an imaging system.

### 4.10 The Phenotype Observation of *ZjSPL3* Homologue Mutants in *A. thaliana* (*AtSPL2/10/11*) and Luciferase Complementation Assay Between zju‐miR157 and ZjSPL3

Seeds of *A. thaliana* mutants corresponding to *ZjSPL3* homologues (*AtSPL2*, *AtSPL10* and *AtSPL11*) were germinated and grown under standard growth conditions. The identification of *AtSPL2*, *AtSPL10* and *AtSPL11* was performed by homologous BLAST method. Phenotypic traits of the mutant lines were compared with wild‐type plants, focusing on developmental differences in shoot branching, leaf morphology and flowering time. For the luciferase complementation assay, the *ZjSPL3* coding sequence was cloned into the pmirGLO dual‐luciferase vector, and *zju‐miR157* was cloned into the *35S::GFP* overexpression vector. These constructs were co‐infiltrated into leaves of 4‐ to 6‐week‐old *N. benthamiana* plants using needleless syringe infiltration. At least three leaves per plant were infiltrated. Three days post‐infiltration, fluorescence signals were detected using an intra‐vital imaging system, as described in Wang, Bai, et al. (2024); Wang, Luo, et al. (2024), to assess the interaction between zju‐miR157 and ZjSPL3.

### 4.11 Heatmap Construction

Heatmaps of DEGs and DAMs were generated using TBtools software, following standardised data visualisation workflows (Chen et al. 2020).

### 4.12 Statistical Analysis

All experiments were conducted using three independent biological replicates, and each experiment was repeated at least three times. For the phenotypes observations were done three times at different time points. For the gene expression, they were repeated for four to five times. For the RNA‐seq, three biological samples were selected from three different trees for each time point. Statistical analysis was performed using one‐way ANOVA in SPSS v. 25.0. Results are presented as mean ± standard error (SE). Differences were considered significant at *p* < 0.05, denoted by *, *p* < 0.01 by ** and *p* < 0.001 by ***. Graphs were generated using GraphPad Prism v. 8.02.

## Author Contributions

**Changfeng Ai:** methodology, software, writing – original draft. **Lixin Wang:** methodology, software, writing – original draft, writing – review and editing. **Zhi Luo:** methodology, software, writing – original draft. **Yunjie Wang:** methodology, software. **Xuan Zhao:** methodology, software. **Lili Wang:** methodology. **Jiaqi Sun:** methodology. **Kunyi Lv:** methodology. **Xueqing Yan:** methodology. **Haonan Cao:** methodology. **Noor Muhammad:** methodology. **Qiong Zhang:** methodology, writing – original draft, writing – review and editing, conceptualization. **Mengjun Liu:** conceptualization. **Zhiguo Liu:** methodology, software, writing – original draft, writing – review and editing, conceptualization.

## Funding

This work was supported by Agricultural Science and Technology Innovation Project of Shandong Academy of Agricultural Sciences (CXGC2025B04). National Natural Science Foundation of China (32471910). Natural Science Foundation of Hebei Province (C2024204016). Key Technology Innovation and Demonstration Project of Forestry and Grassland in Hebei Province (2401096). Hebei Agriculture Research System (HBCT2024190201, HBCT2024190203). Key R&D Program of Shandong Province, China (2025LZGC028).

## Conflicts of Interest

The authors declare no conflicts of interest.

## Supporting information


**Figure S1:** mpp70219‐sup‐0001‐FigureS1.docx.

**Figure S2:** mpp70219‐sup‐0002‐FigureS2.docx.

**Figure S3:** mpp70219‐sup‐0003‐FigureS3.docx.

**Figure S4:** mpp70219‐sup‐0004‐FigureS4.docx.

**Figure S5:** mpp70219‐sup‐0005‐FigureS5.docx.

**Figure S6:** mpp70219‐sup‐0006‐FigureS6.docx.

**Figure S7:** mpp70219‐sup‐0007‐FigureS7.docx.

**Figure S8:** mpp70219‐sup‐0008‐FigureS8.docx.

**Table S1:** mpp70219‐sup‐0009‐TableS1.xlsx.

**Table S2:** mpp70219‐sup‐0010‐TableS2.xlsx.

**Table S3:** mpp70219‐sup‐0011‐TableS3.xlsx.

**Table S4:** mpp70219‐sup‐0012‐TableS4.xlsx.

**Table S5:** mpp70219‐sup‐0013‐TableS5.xlsx.

**Table S6:** mpp70219‐sup‐0014‐TableS6.xlsx.

**Table S7:** mpp70219‐sup‐0015‐TableS7.xlsx.

**Table S8:** mpp70219‐sup‐0016‐TableS8.xlsx.

**Table S9:** mpp70219‐sup‐0017‐TableS9.xlsx.

**Table S10:** mpp70219‐sup‐0018‐TableS10.xlsx.

**Table S11:** mpp70219‐sup‐0019‐TableS11.xlsx.

**Table S12:** mpp70219‐sup‐0020‐TableS12.xlsx.

**Table S13:** mpp70219‐sup‐0021‐TableS13.xlsx.

**Table S14:** mpp70219‐sup‐0022‐TableS14.xlsx.

**Table S15:** mpp70219‐sup‐0023‐TableS15.xlsx.

## Data Availability

Raw transcriptome and metabolome sequence data were deposited in the NGDC database (https://ngdc.cncb.ac.cn) with Accession No. PRJCA047595 and No. PRJCA047653, respectively. The data underlying this article are available in the article and in Figures S1–, S8 and Tables S1–, S15. The corresponding authors were responsible for them.
