# Supplementary material for: Molecular Mechanism of ZjWRKY40‐zju‐miR157 Module Regulating Phytoplasma Tolerance in Jujube
Source: Mol Plant Pathol. 2026 Feb 13;27(2):e70219. doi: 10.1111/mpp.70219 (PMC12904606; doi:10.1111/mpp.70219)
Supplement: Supplementary file 1 — Figure S1: mpp70219‐sup‐0001‐FigureS1.docx. [file MPP-27-e70219-s010.docx]

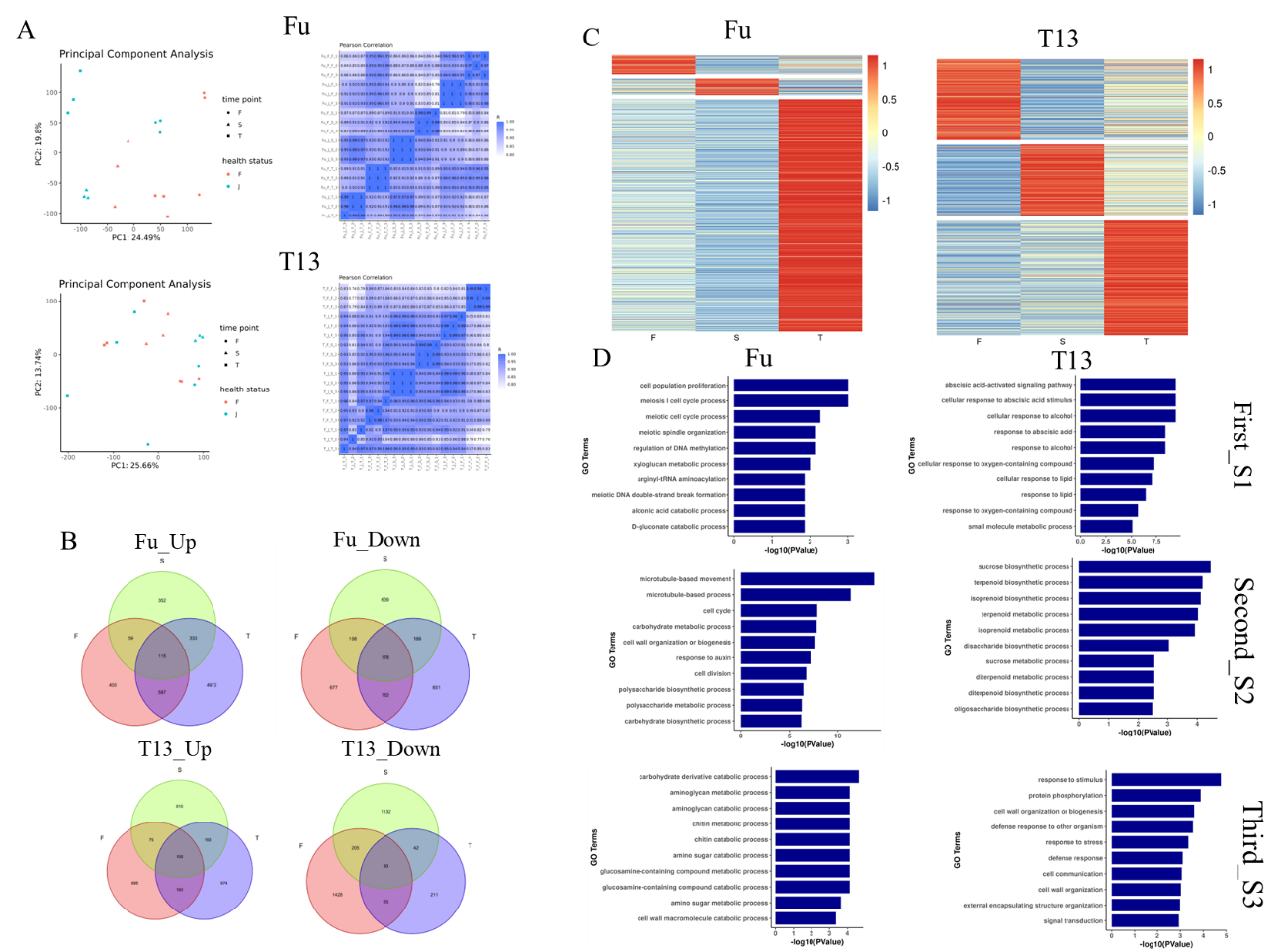


Supplementary Figure S1. Transcriptomic alterations of phytoplasma susceptible genotype Fu and tolerant one T13 under phytoplasma infection at three growth stages. (A) Principal component and pearson correlation analysis of all the samples. (B) Veen diagram analysis of up and down regulated DEGs in Fu and T13 genotypes under phytoplasma infection. (C) Hierarchical clustering heatmap of the fold changes of the transcripts in Fu and T13 diseased plants comparing to their healthy control. (D) Gene ontology enrichment analysis of biological processes about the DEGs at three growth stages in Fu and T13 genotypes.
