# Supplementary material for: Molecular Mechanism of ZjWRKY40‐zju‐miR157 Module Regulating Phytoplasma Tolerance in Jujube
Source: Mol Plant Pathol. 2026 Feb 13;27(2):e70219. doi: 10.1111/mpp.70219 (PMC12904606; doi:10.1111/mpp.70219)
Supplement: Supplementary file 3 — Figure S3: mpp70219‐sup‐0003‐FigureS3.docx. [file MPP-27-e70219-s018.docx]

**
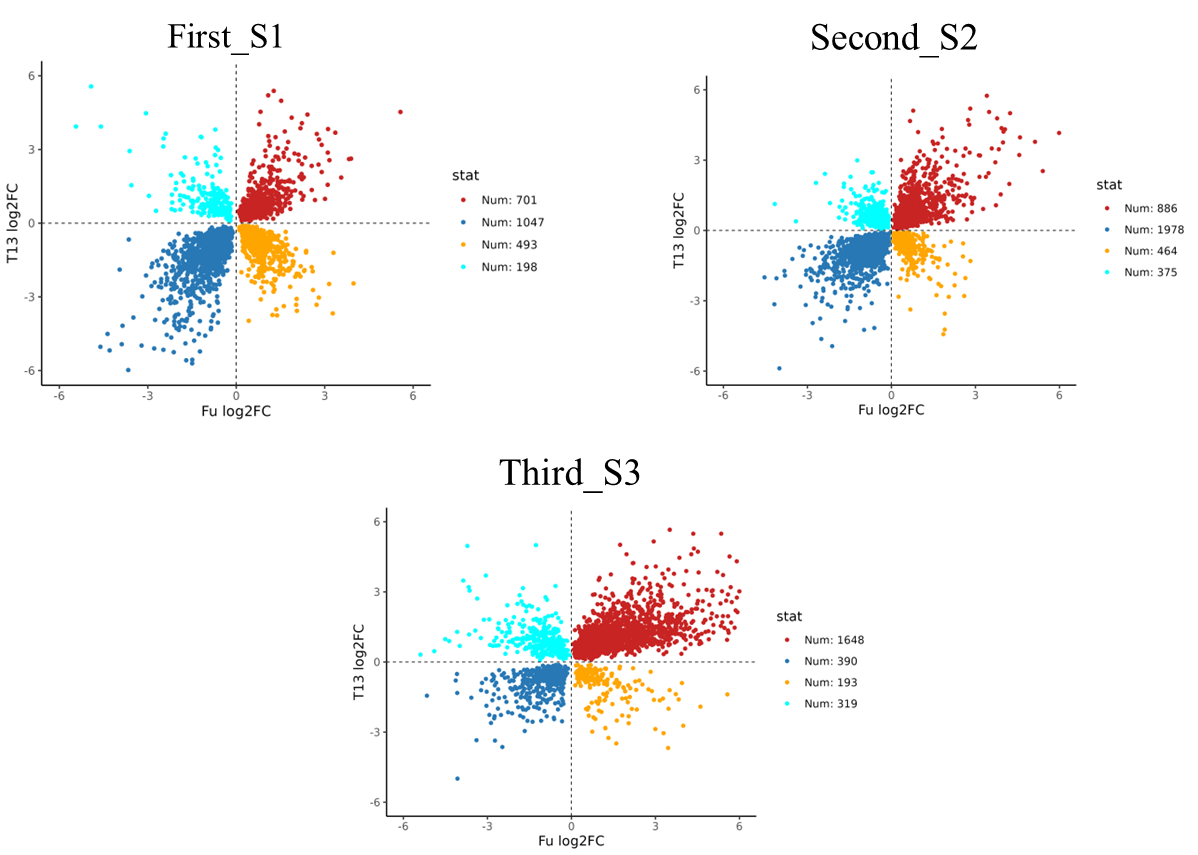
**

Supplementary Figure S3. The scatterplot analysis of DEGs at pvalue_cutoff_0.05 level by Log2FC analysis and between Fu and T13 at three growth stages.
