# Supplementary figures and images for: Molecular Mechanism of ZjWRKY40‐zju‐miR157 Module Regulating Phytoplasma Tolerance in Jujube
Source: Mol Plant Pathol. 2026 Feb 13;27(2):e70219. doi: 10.1111/mpp.70219 (PMC12904606; doi:10.1111/mpp.70219)

**
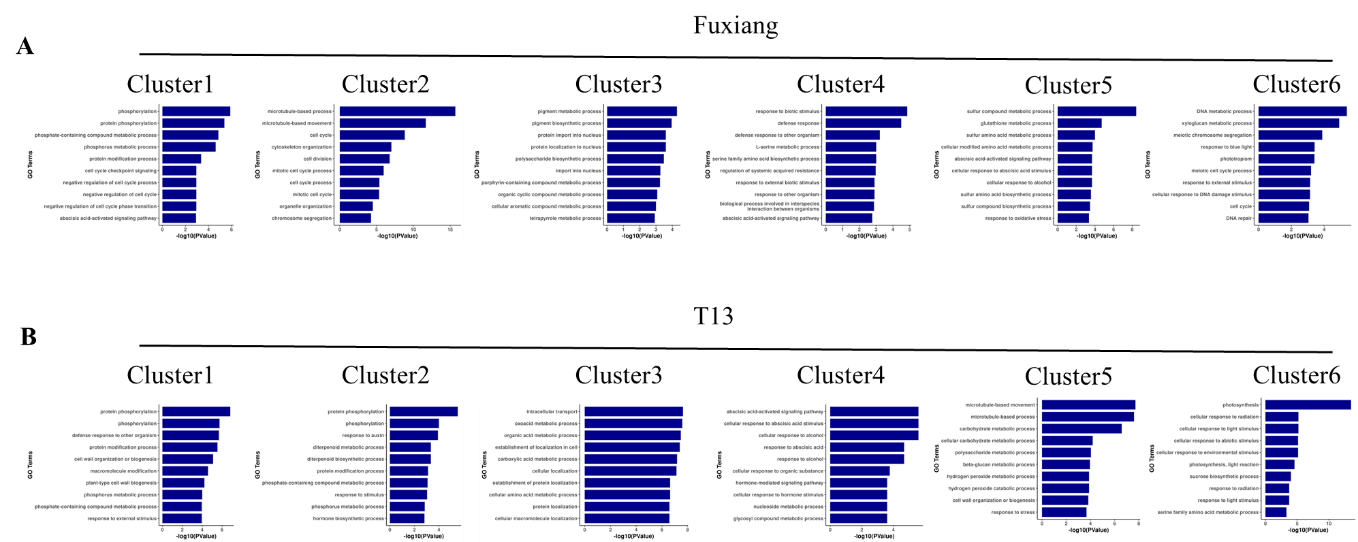
**

Supplementary Figure S4. The GO annotation analysis of the DEGs in six clusters in Fu (A) and T13 (B).

Supplement: Supplementary file 4 — Figure S4: mpp70219‐sup‐0004‐FigureS4.docx. [file MPP-27-e70219-s007.docx]

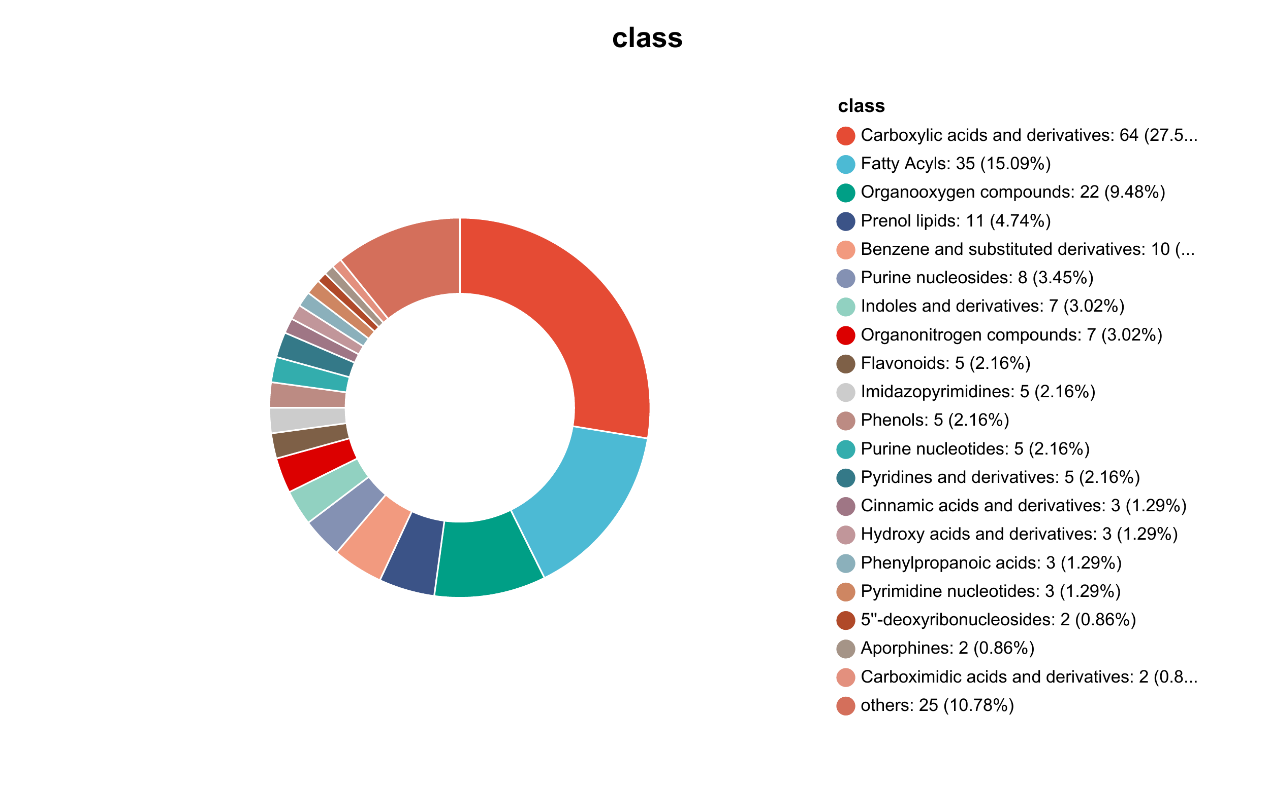


Supplementary Figure S6. The HMDB compound classification analysis.

Supplement: Supplementary file 6 — Figure S6: mpp70219‐sup‐0006‐FigureS6.docx. [file MPP-27-e70219-s023.docx]
