# Supplementary material for: Molecular Mechanism of ZjWRKY40‐zju‐miR157 Module Regulating Phytoplasma Tolerance in Jujube
Source: Mol Plant Pathol. 2026 Feb 13;27(2):e70219. doi: 10.1111/mpp.70219 (PMC12904606; doi:10.1111/mpp.70219)
Supplement: Supplementary file 5 — Figure S5: mpp70219‐sup‐0005‐FigureS5.docx. [file MPP-27-e70219-s008.docx]

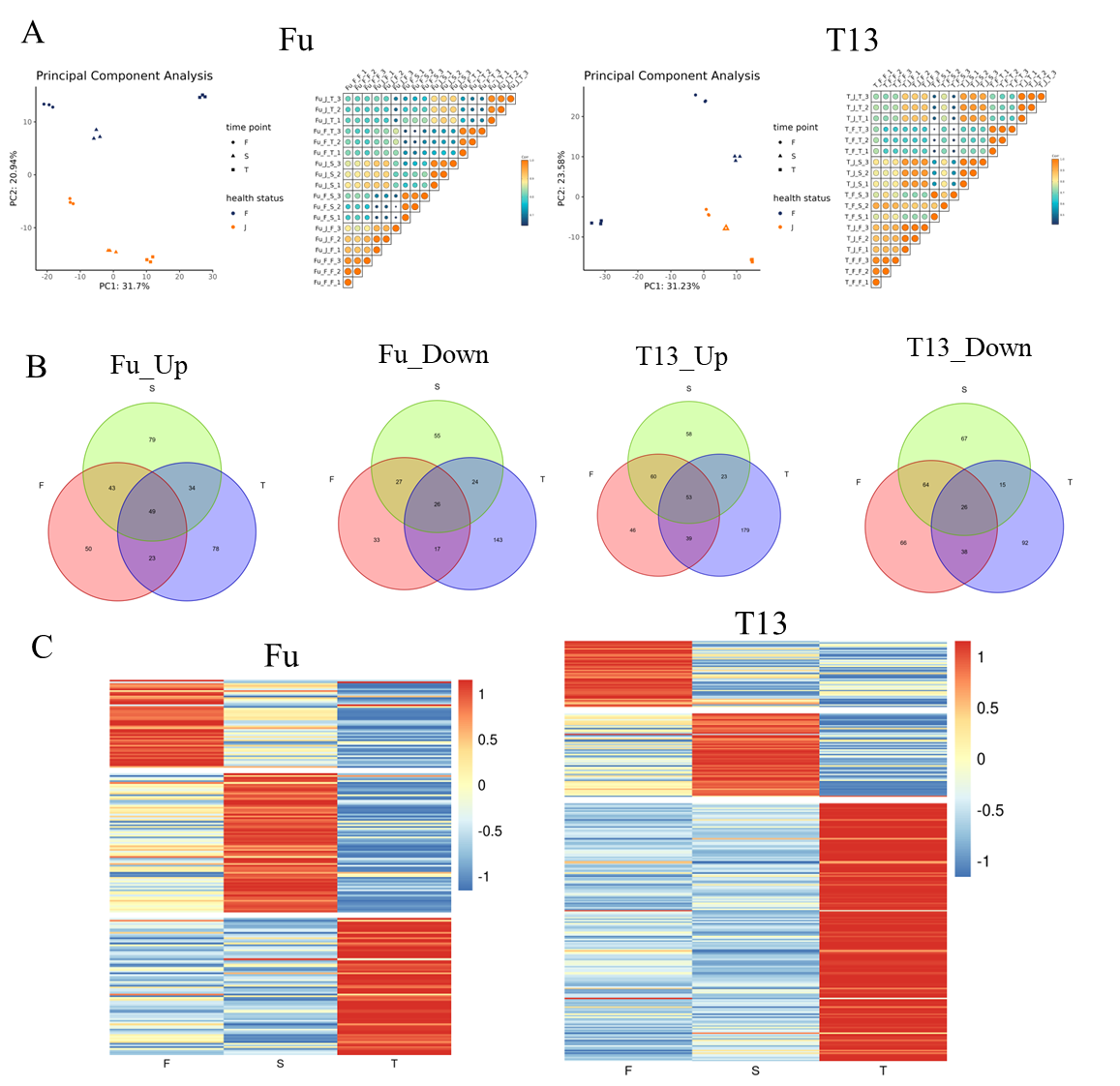


Supplementary Figure S5. (A) Principal component and pearson correlation analysis of all the Fu and T13 samples. (B) Veen diagram analysis of up and down regulated DAMs in Fu and T13 under phytoplasma infection. (C) Hierarchical clustering heatmap of the fold changes of metabolites in Fu and T13 diseased plants comparing to their healthy control.
