# Supplementary material for: Molecular Mechanism of ZjWRKY40‐zju‐miR157 Module Regulating Phytoplasma Tolerance in Jujube
Source: Mol Plant Pathol. 2026 Feb 13;27(2):e70219. doi: 10.1111/mpp.70219 (PMC12904606; doi:10.1111/mpp.70219)
Supplement: Supplementary file 7 — Figure S7: mpp70219‐sup‐0007‐FigureS7.docx. [file MPP-27-e70219-s017.docx]

**
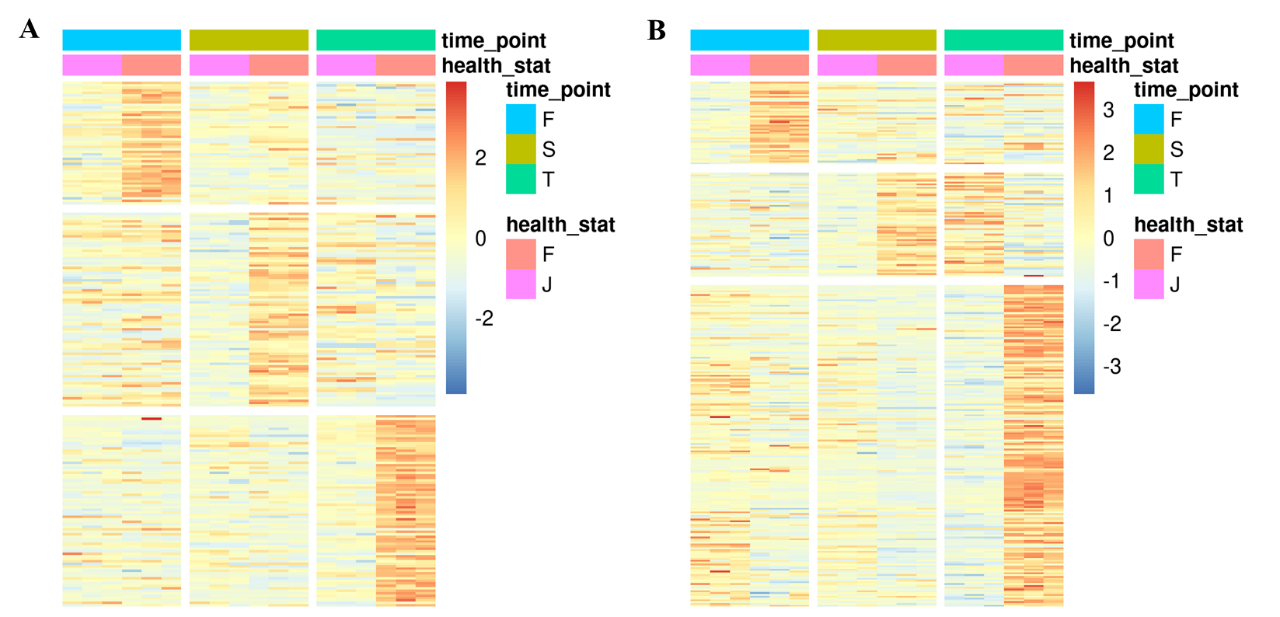
**

Supplementary Figure S7. The heatmap of DAMs in Fu (A) and T13 (B) diseased plants and healthy control plants at three growth stages.
