# Supplementary material for: Molecular Mechanism of ZjWRKY40‐zju‐miR157 Module Regulating Phytoplasma Tolerance in Jujube
Source: Mol Plant Pathol. 2026 Feb 13;27(2):e70219. doi: 10.1111/mpp.70219 (PMC12904606; doi:10.1111/mpp.70219)
Supplement: Supplementary file 8 — Figure S8: mpp70219‐sup‐0008‐FigureS8.docx. [file MPP-27-e70219-s002.docx]

>SJP4_JWB_

MFKLKKQLYLFKIVLFICLGLLFVINNNNNQVMAMENSKTIQEQKEERIRKNHELVQNKIIIINENLEKREQLEKQIEELKSQPKNKKTNKEIANLEKEIINCTHFIGFHRNQIKMIRRYG

>SJP1_JWB_

MVKLQNQFKIISICLFTFLGLFLITNNVHQIMAAPKKNHGKDIISSKEESKKDVKNFYELHNTLENYSEEDRNKIIQMLSNPEITKILEKKAKETKTQEKGSSSKKPNKKQK

>SJP2_JWB_

MLKFKHNLFFLNIFLFIILGMFLITNNFHQIMAASKNNGKDIISSKEEAKNNVKKYYELYNTLENYSEEERNKIIQMLSNPEIIKTLEEKIKETKTQEKGSFSKKPDNLKK

>SJP3_JWB_

MMQIKNKLHLLPLFLMSFLGLFAFININPVMATDPKLPETSSRQPVNQNFTIEENIINLKQKIYDNATKITNIDKELQGSITDNQKENLLKLKENYKQLIDNQKEQLKTYKNLLNNLNDENN

>PHYL1

MMQIKNKLHLLPLFLMSFLGLFAFININPVMATDPKLPETSSRQPVNQNFTIEENIINLKQKIYDNATKITNIDKELQGSITDNQKENLLKLKENYKQLIDNQKEQLKTYKNLLKNLNDENN

>Zaofeng3

MMQIKNKLHLLPLFLMSFLGLFAFININPVMATDPKLPETSSRQPVNQNFTIEENIINLKQKIYDNATKITNIDKELQGSITDNQKENLLKLKENYKQLIDNQKEQLKTYKNLLNNLNDENN

>Zaofeng6

MLKFKHNLFFLNIFLFIILGMFLITNNFHQIMAASKNNGKDIISSKEEAKNNVKKYYELYNTLENYSEEERNKIIQMLSNPEIIKTLEEKIKETKTQEKGSFSKKPDNLKK


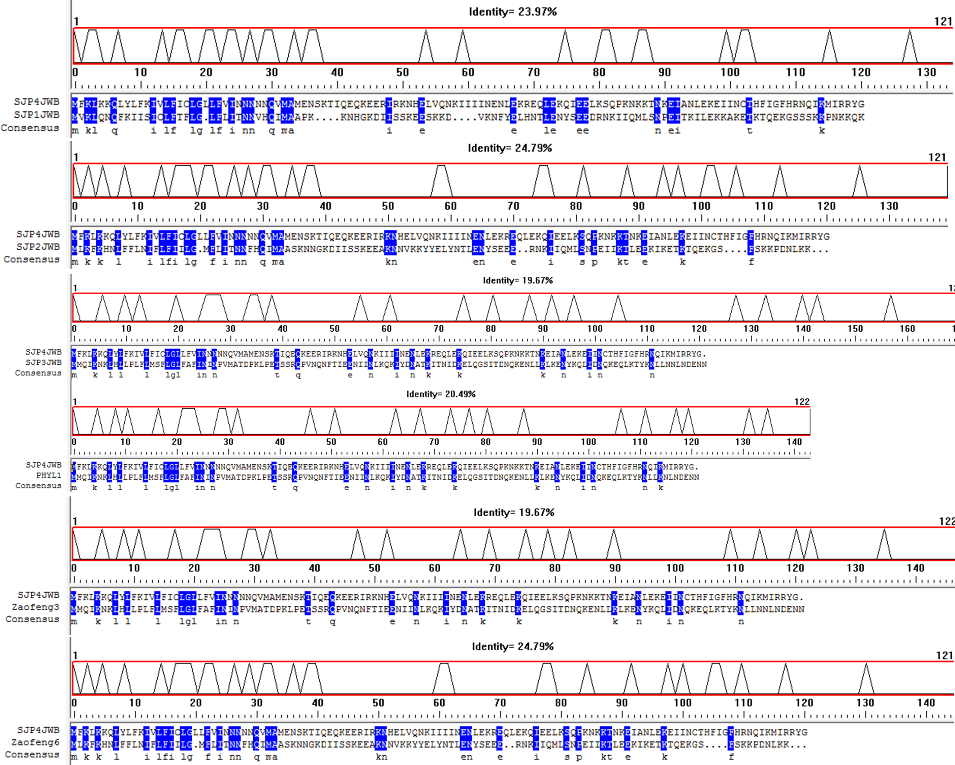


Supplementary Figure S8. The character of SJP4_JWB_.
